# The limits of fair medical imaging AI in real-world generalization

**DOI:** 10.1038/s41591-024-03113-4

**Published:** 2024-06-28

**Authors:** Yuzhe Yang, Haoran Zhang, Judy W. Gichoya, Dina Katabi, Marzyeh Ghassemi

**Affiliations:** 1 Department of Electrical Engineering and Computer Science, Massachusetts Institute of Technology, Cambridge, MA USA; 2 Department of Radiology, Emory University School of Medicine, Atlanta, GA USA; 3 Institute for Medical Engineering & Science, Massachusetts Institute of Technology, Cambridge, MA USA

**Keywords:** Diagnosis, Radiography

## Abstract

As artificial intelligence (AI) rapidly approaches human-level performance in medical imaging, it is crucial that it does not exacerbate or propagate healthcare disparities. Previous research established AI’s capacity to infer demographic data from chest X-rays, leading to a key concern: do models using demographic shortcuts have unfair predictions across subpopulations? In this study, we conducted a thorough investigation into the extent to which medical AI uses demographic encodings, focusing on potential fairness discrepancies within both in-distribution training sets and external test sets. Our analysis covers three key medical imaging disciplines—radiology, dermatology and ophthalmology—and incorporates data from six global chest X-ray datasets. We confirm that medical imaging AI leverages demographic shortcuts in disease classification. Although correcting shortcuts algorithmically effectively addresses fairness gaps to create ‘locally optimal’ models within the original data distribution, this optimality is not true in new test settings. Surprisingly, we found that models with less encoding of demographic attributes are often most ‘globally optimal’, exhibiting better fairness during model evaluation in new test environments. Our work establishes best practices for medical imaging models that maintain their performance and fairness in deployments beyond their initial training contexts, underscoring critical considerations for AI clinical deployments across populations and sites.

## Abstract (web summary)

When tested across tasks, diseases and imaging modalities, the performance of AI models depends on encoding of demographic shortcuts, and correcting for them decreases their ability to generalize in new populations.

## Main

As artificial intelligence (AI) models are increasingly deployed in real-world clinical settings^1,2^, it is crucial to evaluate not only model performance but also potential biases toward specific demographic groups^3,4^. Although deep learning has achieved human-level performance in numerous medical imaging tasks^5,6^, existing literature indicates a tendency for these models to manifest existing biases in the data, causing performance disparities between protected subgroups^7–11^. For instance, chest X-ray (CXR) classifiers trained to predict the presence of disease systematically underdiagnose Black patients^12^, potentially leading to delays in care. To ensure the responsible and equitable deployment of such models, it is essential to understand the source of such biases and, where feasible, take actions to correct them^13,14^.

Recent studies have unveiled the surprising ability of deep models to predict demographic information, such as self-reported race^15^, sex and age^16^, from medical images, achieving performance far beyond that of radiologists. These insights raise the concern of disease prediction models leveraging demographic features as heuristic ‘shortcuts’^17,18^—correlations that are present in the data but have no real clinical basis^18^, for instance deep models using the hospital as a shortcut for disease prediction^19,20^.

In this work, we investigated four questions. First, we consider whether disease classification models also use demographic information as shortcuts and whether such demographic shortcuts result in biased predictions. Second, we evaluate the extent to which state-of-the-art methods can remove such shortcuts and create ‘locally optimal’ models that are also fair. Third, we consider real-world clinical deployment settings where shortcuts may not be valid in the out-of-distribution (OOD) data, to dissect the interplay between algorithmic fairness and shortcuts when data shift. Finally, we explore which algorithms and model selection criteria can lead to ‘globally optimal’ models that maintain fairness when deployed in an OOD setting.

**Extended Data Fig. 1:**
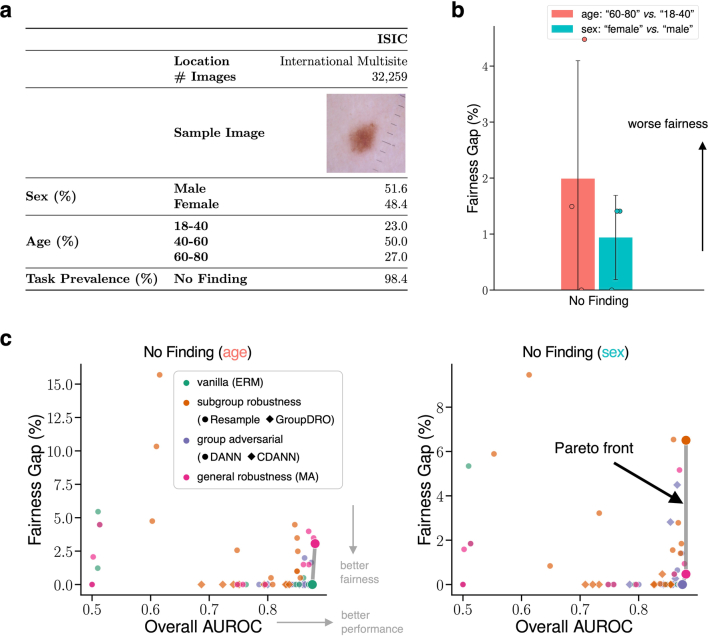
Evaluation results for models on the ISIC dataset for No Finding prediction. **a**, Dataset statistics. **b**, Subgroup fairness gaps of the ERM model as defined by FPR. Each subgroup contains at least 100 samples for analysis (age: subgroup ‘60–80’ vs ‘18–40’; sex: subgroup ‘female’ vs ‘male’). We find that disparities in FPR are small and statistically insignificant in the case of age. Each bar and its error bar indicate the mean and standard deviation across 3 independent runs. **c**, Trade-off between the fairness gap and overall AUROC for all trained models, evaluated against sensitive attribute age and sex, respectively. We find that most models, including ERM, achieve a good fairness-performance tradeoff.

**Extended Data Fig. 2:**
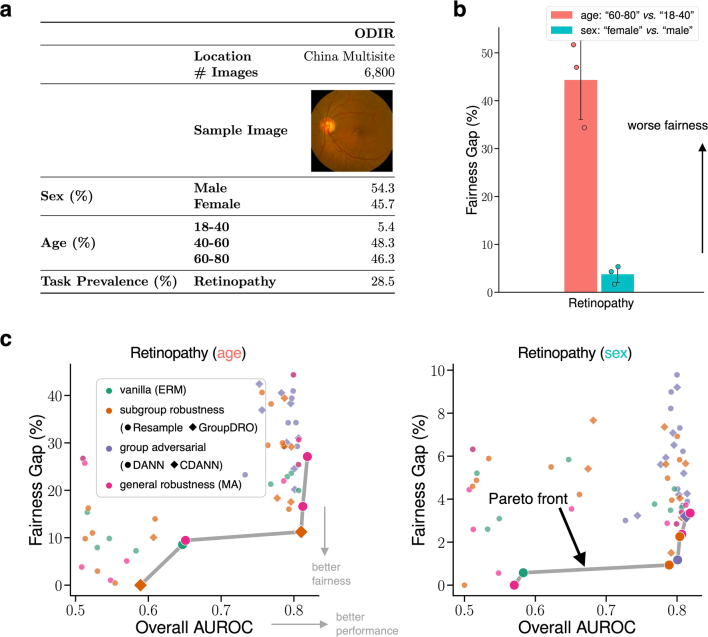
Evaluation results for models on the ODIR dataset for Retinopathy prediction. **a**, Dataset statistics. **b**, Subgroup fairness gaps of the ERM model as defined by FNR. Each subgroup contains at least 100 samples for analysis (age: subgroup ‘60–80’ vs ‘18–40’; sex: subgroup ‘female’ vs ‘male’). We find a significant FNR gap between age groups. Each bar and its error bar indicate the mean and standard deviation across 3 independent runs. **c**, Trade-off between the fairness gap and overall AUROC for all trained models, evaluated against sensitive attribute age and sex, respectively. We find, similar to the chest X-ray setting, that algorithms for fixing demographic shortcuts could improve in-distribution fairness while incurring minimal detriments to performance.

We performed a systematic investigation into how medical AI leverages demographic shortcuts through these questions, with an emphasis on fairness disparities across both in-distribution (ID) training and external test sets. Our primary focus is on CXR prediction models, with further validation in dermatology (Extended Data Fig. 1) and ophthalmology (Extended Data Fig. 2). Our X-ray analysis draws upon six extensive, international radiology datasets: MIMIC-CXR^21^, CheXpert^22^, NIH^23^, SIIM^24^, PadChest^25^ and VinDr^26^. We explored fairness within both individual and intersectional subgroups spanning race, sex and age^12^. Our assessment uncovers compelling new insights into how medical AI encodes demographics and the impact that this has on various fairness considerations, especially when models are applied outside their training context during real-world domain shifts, with actionable insights on what models to select for fairness under distribution shift.

## Results

### Datasets and model training

We used six publicly available CXR datasets, as described in Table 1. We focused on four binary classification tasks that have been shown to have disparate performance between protected groups^7,27^: ‘No Finding’, ‘Effusion’, ‘Pneumothorax’ and ‘Cardiomegaly’. The detailed prevalence rates of the diseases for each demographic subgroup are shown in Extended Data Table 1.

**Table 1:** Demographic and label characteristics of the six X-ray datasets used in this study

		MIMIC	CheXpert	NIH	SIIM	PadChest	VinDr
	Location	Boston, MA	Stanford, CA	Bethesda, MD	Bethesda, MD	Alicante, Spain	Hanoi, Vietnam
	No. of images	357,167	222,792	112,120	11,582	144,478	6,354
	Percent frontal	64.5	85.5	100.0	100.0	69.1	100.0
Sex (%)	Female	47.8	40.7	43.5	44.6	50.4	43.1
	Male	52.2	59.3	56.5	55.4	49.6	56.9
Race (%)	Asian	3.1	10.5	–	–	–	–
	Black	15.6	5.4	–	–	–	–
	White	61.0	56.4	–	–	–	–
	Other	20.3	27.8	–	–	–	–
Age (%)	0–18	–	–	4.8	5.0	3.7	21.8
	18–40	13.8	13.9	27.7	27.3	9.2	16.0
	40–60	31.1	31.1	43.9	42.9	26.5	27.1
	60–80	40.0	39.0	22.7	23.9	38.0	30.0
	80–100	15.1	16.0	0.9	0.9	22.6	5.1
Intersection (%)	Asian female	1.5	4.5	–	–	–	–
	Asian male	1.6	6.0	–	–	–	–
	Black female	9.3	2.6	–	–	–	–
	Black male	6.3	2.7	–	–	–	–
	White female	27.3	22.2	–	–	–	–
	White male	33.8	34.1	–	–	–	–
	Others female	9.8	11.3	–	–	–	–
	Others male	10.5	16.5	–	–	–	–
Task prevalence (%)	No Finding	39.8	10.0	53.8	–	34.9	41.2
	Effusion	20.0	38.6	11.9	–	5.9	7.5
	Pneumothorax	3.4	8.7	4.7	28.4	0.3	0.7
	Cardiomegaly	14.9	12.1	2.5	–	9.5	22.6

We also examined medical AI applications in dermatology and ophthalmology. Specifically, we used the ISIC dataset^28^ with ‘No Finding’ as the task for dermatological imaging (Extended Data Fig. 1a) and the ODIR dataset^29^ with ‘Retinopathy’ as the task for ophthalmology images (Extended Data Fig. 2a).

To evaluate fairness, we examined the class-conditioned error rate that is likely to lead to worse patient outcomes for a screening model. For ‘No Finding’, a false positive indicates falsely predicting that a patient is healthy when they are ill, which could lead to delays in treatment^12^; we, therefore, evaluated the differences in false-positive rate (FPR) between demographic groups. For all other diseases, we evaluated the false-negative rate (FNR) for the same reason. Equality in these metrics is equivalent to equality of opportunity^30^. We choose to study fairness through the notion of equalized odds, as it has been widely used in previous work in the CXR and fairness literature^7,12^. In addition, shortcut learning using a particular demographic attribute leads to differences in class-conditioned error rates (that is, FPR and FNR gaps) across attributes^31,32^, and so studying these gaps allows us to glean insight into the severity of shortcut learning. Finally, FPR and FNR (as enforced to be equal by equalized odds) are meaningful metrics in the clinical setting, as they correspond to error rates of decision-making at the individual level^12^.

To understand and quantify the types and degrees of distribution shifts in our study, we examined whether there are significant statistical differences in distributions between demographic groups in the ID settings as well as across different datasets in the OOD settings. Specifically, we analyzed prevalence shifts P(Y|A) and representation shifts P(X|A) across different subgroups for ID scenarios and added label shifts P(Y) and covariate shifts P(X) for OOD scenarios (Methods). Our analyses indicate that all the distributions that we examined show statistically significant shifts, affecting most demographic groups in the ID context (Extended Data Table 2) and across various sites in the OOD context (Extended Data Table 3). We note that our analysis does not presuppose specific types of distribution shifts; instead, we simulated real-world deployment conditions where any of these shifts might occur, aiming for results that are generalizable to complex, real-world scenarios.

**Fig. 1:**
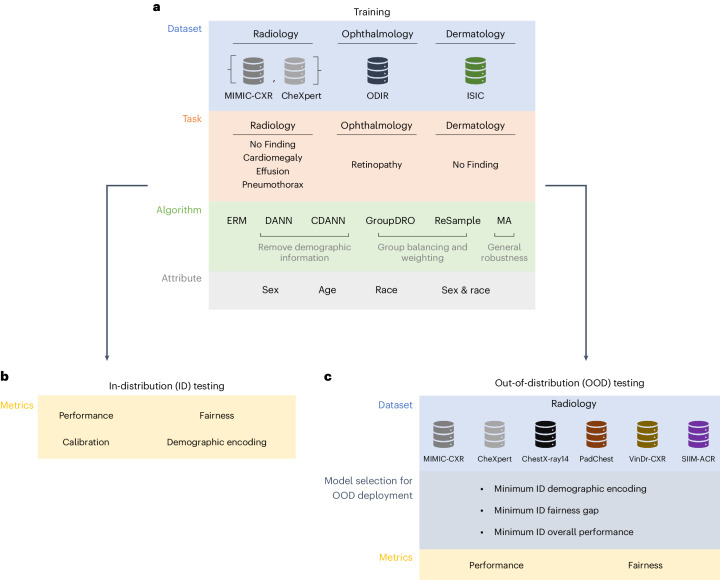
Overall experimental pipeline. **a**, We trained a grid of deep learning models on medical images from a variety of modalities on several clinical tasks. We applied a variety of state-of-the-art algorithms to mitigate shortcuts, for up to four demographic attributes (where available). **b**, We evaluated each model ID (that is, on the same dataset where it is trained), along the axis of performance, fairness, amount of demographic encoded and calibration. **c**, We evaluated the performance and fairness of CXR classification models on OOD domains. To mimic a realistic deployment setting where OOD samples are not observed, we chose the ‘best’ model based on several ID selection criteria.

We trained a grid of deep convolutional neural networks^33^ on MIMIC-CXR (radiology), CheXpert (radiology), ODIR (ophthalmology) and ISIC (dermatology), varying the classification task. Our approach follows previous work that achieves state-of-the-art performance in these tasks^8,12^ using empirical risk minimization (ERM)^34^. We also evaluated algorithms designed to remove spurious correlations or increase model fairness during training. We categorized these algorithms into those that (1) reweight samples based on their group to combat underrepresentation (ReSample^35^ and GroupDRO^36^); (2) adversarially remove group information from model representations (DANN^37^ and CDANN^38^); and (3) more generically attempt to improve model generalization—that is, exponential moving average (MA^39^). In total, our analysis encompassed a total of 3,456 models trained on MIMIC-CXR, corresponding to the cartesian product of four tasks, four demographic attributes, six algorithms, 12 hyperparameter settings and three random seeds. We summarized our experimental pipeline in Fig. 1.

### Algorithmic encoding of attributes leads to fairness gaps

**Fig. 2:**
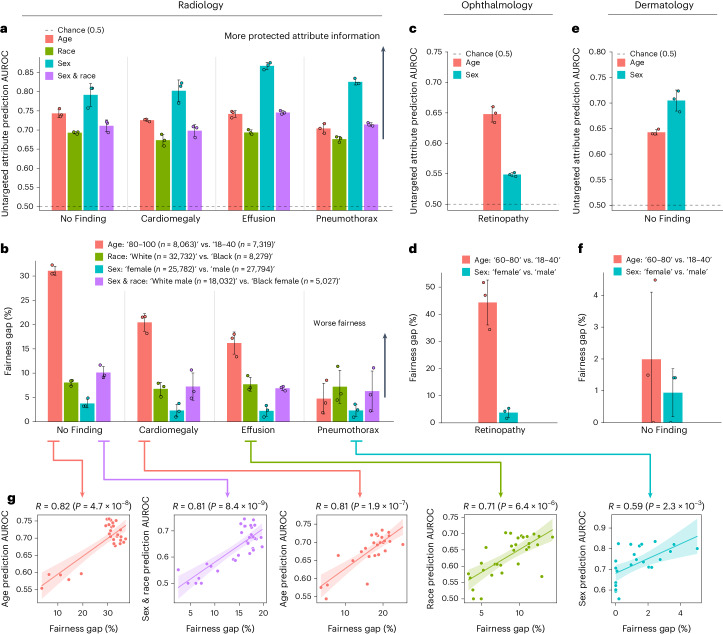
Medical imaging models encode sensitive attributes and are unfair across subgroups. **a**, The AUROC of demographic attribute prediction from frozen representations for the best ERM model. We trained ERM models on MIMIC-CXR to predict four different binary tasks. ERM representations encode demographic attributes to a high degree. **b**, The fairness gap, as defined by the FPR gap for ‘No Finding’, and the FNR gap for all other tasks for the best ERM model. ERM models exhibit high fairness gaps, especially between age groups. **c**, The AUROC of demographic attribute prediction from frozen representations for the best ERM model on the ODIR dataset (ophthalmology), following the same experimental setup. **d**, The fairness gap for the best ERM model on the ODIR dataset (ophthalmology). **e**, The AUROC of demographic attribute prediction from frozen representations for the best ERM model on the ISIC dataset (dermatology), following the same experimental setup. **f**, The fairness gap for the best ERM model on the ISIC dataset (dermatology). **a**–**f**, Each bar and its error bar indicate the mean and standard deviation across three independent runs. **g**, The correlation between attribute prediction performance and fairness for all learned models. We excluded models with suboptimal performance—that is, with an overall validation AUROC below 0.7. The attribute prediction AUROC shows a high correlation with the fairness gap (‘No Finding’, age: *R* = 0.82, *P* = 4.7 × 10^−8^; ‘No Finding’, sex and race: *R* = 0.81, *P* = 8.4 × 10^−9^; ‘Cardiomegaly’, age: *R* = 0.81, *P* = 1.9 × 10^−7^; ‘Effusion’, race: *R* = 0.71, *P* = 6.4 × 10^−6^; ‘Pneumothorax’, sex: *R* = 0.59, *P* = 2.3 × 10^−3^; all using two-sided *t*-test). The center line and the shadow denote the mean and 95% CI, respectively.

We separately trained deep learning models for our four distinct CXR prediction tasks (‘No Finding’, ‘Cardiomegaly’, ‘Effusion’ and ‘Pneumothorax’) as well as ‘Retinopathy’ in ophthalmology and ‘No Finding’ in dermatology. Each model consists of a feature extractor followed by a disease prediction head. We then employed a transfer learning approach, wherein we kept the weights of the feature extractor frozen and retrained the model to predict sensitive attributes (for example, race). This allowed us to assess the amount of attribute-related information present in the features learned by each model as measured by the area under the receiver operating characteristic curve (AUROC) for attribute prediction (Methods). Previous work^15,40^ demonstrated that deep models trained for disease classification encode demographic attributes, and such encoding could lead to algorithmic bias^41^. We extend the investigation to a broader array of datasets, attributes and imaging modalities. As Fig. 2a,c,e confirms, the penultimate layer of different disease models contains significant information about four demographic attributes (age, race, sex and the intersection of sex and race), and that is consistent across different tasks and medical imaging modalities.

**Extended Data Fig. 3:**
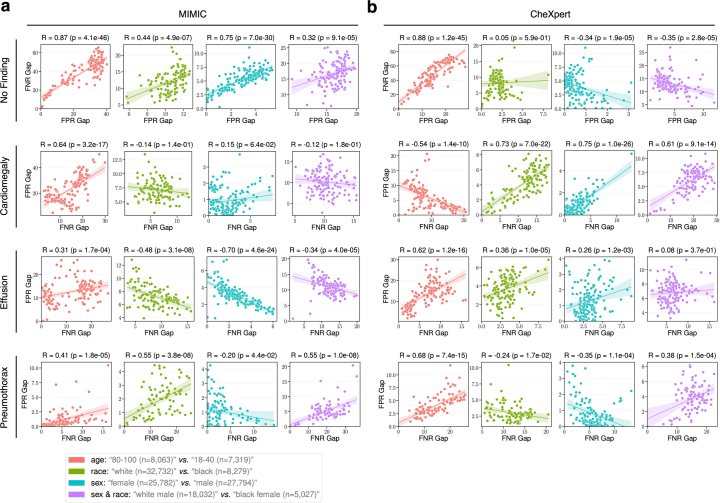
Trade-offs between the FPR gap and the FNR gap for each task and attribute, for models trained on MIMIC-CXR or CheXpert and evaluated on the same dataset for (a) No Finding and (b) Effusion prediction. We evaluate these metrics across age (‘80–100’ vs ‘18–40’), sex (‘female’ vs ‘male’), race (‘White’ vs. ‘Black’), and the intersection of sex and race (‘White male’ vs. ‘Black female’). We find for the most part, there is a positive correlation, indicating that fairer models achieve fairness with respect to both FPR and FNR (that is, equal odds). All p values are calculated using two-sided t-test. The center line and the shadow denote the mean and 95% CI, respectively.

We then assessed the fairness of these models across demographic subgroups as defined by equal opportunity^30^—that is, discrepancies in the model’s FNR or FPR for demographic attributes. We focused on underdiagnosis^12^—that is, discrepancies in FPR for ‘No Finding’ and discrepancies in FNR for other diseases. For each demographic attribute, we identified two key subgroups with sufficient sample sizes: age groups ‘80–100’ (*n* = 8,063) and ‘18–40’ (*n* = 7,319); race groups ‘White’ (*n* = 32,732) and ‘Black’ (*n* = 8,279); sex groups ‘female’ (*n* = 25,782) and ‘male’ (*n* = 27,794); and sex and race groups ‘White male’ (*n* = 18,032) and ‘Black female’ (*n* = 5,027). In all tasks, we observed that the models displayed biased performance within the four demographic attributes, as evidenced by the FNR disparities (Fig. 2b). The observed gaps can be as large as 30% for age. The same results hold for the other two imaging modalities (Fig. 2d,f). Similar results for overdiagnosis (FNR of ‘No Finding’ and FPR for disease prediction) can be found in Extended Data Fig. 3.

We further investigated the degree to which demographic attribute encoding ‘shortcuts’ may impact model fairness. When models use demographic variables as shortcuts, previous work showed that they can exhibit gaps in subgroup FPR and FNR^31,40^. We note that a model encoding demographic information does not necessarily imply a fairness violation, as the model may not necessarily use this information for its prediction. For each task and attribute combination, we trained different models with varying hyperparameters (Methods). We focused on the correlation between the degree of encoding of different attributes and the fairness gaps as assessed by underdiagnosis. Figure 2g shows that a stronger encoding of demographic information is significantly correlated with stronger model unfairness (‘No Finding’, age: *R* = 0.82, *P* = 4.7 × 10^−8^; ‘No Finding’, sex and race: *R* = 0.81, *P* = 8.4 × 10^−9^; ‘Cardiomegaly’, age: *R* = 0.81, *P* = 1.9 × 10^−7^; ‘Effusion’, race: *R* = 0.71, *P* = 6.4 × 10^−6^; ‘Pneumothorax’, sex: *R* = 0.59, *P* = 2.3 × 10^−3^; all using two-sided *t*-test). Such consistent observations indicate that models using demographic encodings as heuristic shortcuts also have larger fairness disparities, as measured by discrepancies in FPR and FNR.

### Mitigating shortcuts creates locally optimal models

**Fig. 3:**
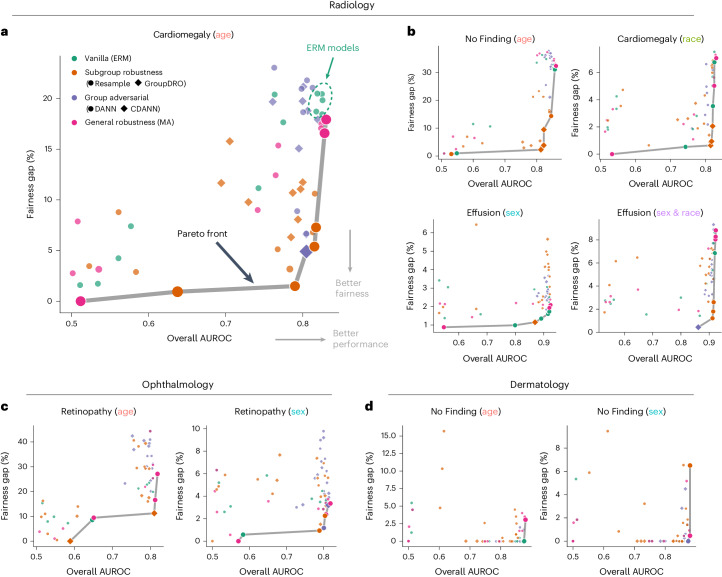
Algorithms for removing demographic shortcuts mitigate ID fairness gaps and maintain performance. **a**, Tradeoff between the fairness gap and overall AUROC for all trained models, for ‘Cardiomegaly’ prediction using ‘age’ as the attribute. We plotted the Pareto front—the best achievable fairness gap with a minimum constraint on the performance. **b**, Tradeoff between the fairness gap and overall AUROC for all trained models, with more disease prediction tasks and attributes. **c**, Tradeoff between the fairness gap and the overall AUROC on the ODIR dataset (ophthalmology). **d**, Tradeoff between the fairness gap and the overall AUROC on the ISIC dataset (dermatology).

**Extended Data Fig. 4:**
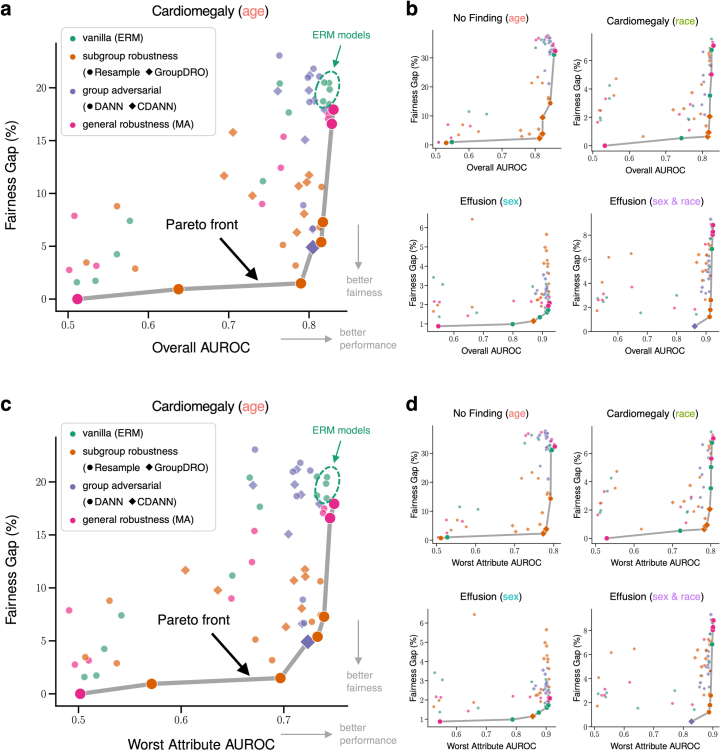
Algorithms for removing demographic shortcuts mitigate in-distribution fairness gaps and maintain performance. **a, b**, Trade-off between the fairness gap and overall AUROC for all trained models. **c, d**, Trade-off between the fairness gap and the worst-group AUROC for all trained models. Each plot represents a specific disease prediction task (for example, Cardiomegaly) with a specific attribute (for example, age). In each case, we plot the Pareto front, the best achievable fairness gap with a minimum constraint on the performance.

We performed model evaluations first in the ID setting, where ERM models trained and tested on data from the same source performed well. We compared ERM to state-of-the-art robustness methods that were designed to effectively address fairness gaps while maintaining overall performance. As shown in Fig. 3a, ERM models exhibited large fairness gaps across age groups when predicting ‘Cardiomegaly’ (that is, models centered in the top right corner, FNR gap of 20% between groups ‘80–100’ and ‘18–40’). By applying data rebalancing methods to address prevalence shifts during training (for example, ReSample), we observed reduced fairness gaps in certain contexts. By applying debiasing robustness methods that correct demographic shortcuts, such as GroupDRO and DANN, the resulting models were able to close the FNR gap while achieving similar AUROCs (for example, the bottom right corner). Our results hold when using the worst group AUROC as the performance metric (Extended Data Fig. 4) and across different combinations of diseases and attributes (Fig. 3b and Extended Data Fig. 4).

To demonstrate the value of model debiasing, we further plotted the set of ‘locally optimal models’—those on the Pareto front^42^ that balance the performance–fairness tradeoff most optimally on ID data (Fig. 3a). Those models that lie on this front are ‘locally optimal’, as they have the smallest fairness gap that can be achieved for a fixed performance constraint (for example, AUROC > 0.8). In the ID setting, we found several existing algorithms that consistently achieve high ID fairness without losing notable overall performance for disease prediction (Fig. 3a,b and Extended Data Fig. 4).

Similar to our observations in radiology, we identified fairness gaps within subgroups based on age and sex in dermatology and ophthalmology, respectively (Fig. 2d,f). We further verified the Pareto front for both attributes, where similar observations hold that algorithms for fixing demographic shortcuts could improve ID fairness while incurring minimal detriments to performance (Fig. 3c,d). The steepness of the Pareto front suggests that small sacrifices in performance could yield substantial gains in fairness.

### Locally optimal models exhibit tradeoffs in other metrics

**Extended Data Fig. 5:**
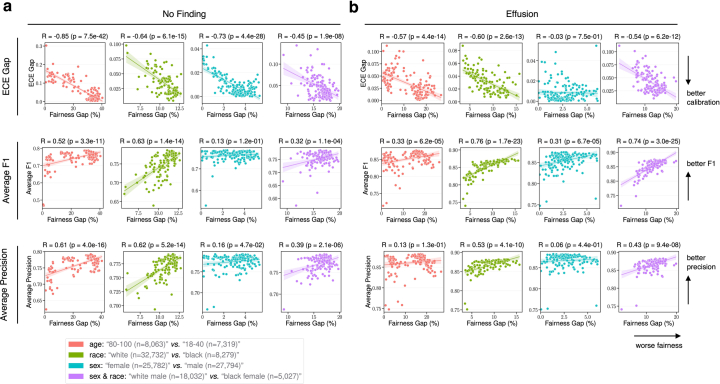
Trade-offs between the fairness gap and the expected calibration error (ECE) gap, average F1, and average precision, for models trained and evaluated on MIMIC-CXR for (a) No Finding and (b) Effusion prediction. We evaluate these metrics across age (‘80–100’ vs ‘18–40’), sex (‘female’ vs ‘male’), race (‘White’ vs. ‘Black’), and the intersection of sex and race (‘White male’ vs. ‘Black female’). We find that fairer models tend to exhibit larger ECE gaps, worse average F1, and worse average precision, indicating the undesirable tradeoff between fairness with other performance and calibration metrics. All p values are calculated using two-sided t-test. The center line and the shadow denote the mean and 95% CI, respectively.

We examined how locally optimal models that balance fairness and AUROC impact other metrics, as previous work showed that it is a theoretical impossibility to balance fairness measured by probabilistic equalized odds and calibration by group^43^. We found that optimizing fairness alone leads to worse results for other clinically meaningful metrics in some cases, indicating an inherent tradeoff between fairness and other metrics. First, for the ‘No Finding’ prediction task, enforcing fair predictions across groups results in worse expected calibration error gap (ECE Gap; Extended Data Fig. 5a) between groups. Across different demographic attributes, we found a consistent statistically significant negative correlation between ECE Gap and Fairness Gap (age: *R* = −0.85, *P* = 7.5 × 10^−42^; race: *R* = −0.64, *P* = 6.1 × 10^−15^; sex: *R* = −0.73, *P* = 4.4 × 10^−28^; sex and race: *R* = −0.45, *P* = 1.9 × 10^−8^; all using two-sided *t*-test).

We explored the relationship between fairness and other metrics, including average precision and average F1 score. For ‘No Finding’ prediction, fairer models lead to both worse average precision and F1 score (Extended Data Fig. 5a). The same trend holds across different diseases—for example, for ‘Effusion’ (Extended Data Fig. 5b). These findings stress that these models, although being locally optimal, exhibit worse results on other important and clinically relevant performance metrics. This uncovers the limitation of blindly optimizing fairness, emphasizing the necessity for more comprehensive evaluations to ensure the reliability of medical AI models.

### Local fairness does not transfer under distribution shift

**Fig. 4:**
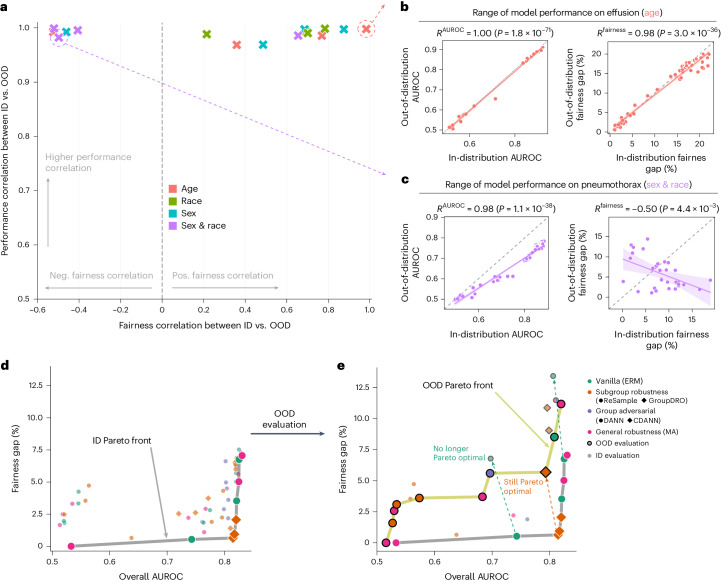
The transfer of performance (overall AUROC) and fairness between the ID (MIMIC-CXR) and OOD datasets. **a**, We plotted the Pearson correlation coefficient of ID versus OOD performance versus the Pearson correlation coefficient of ID versus OOD fairness. Here, each point was derived from a grid of models trained on a particular combination of task and attribute. We found that there was a high correlation between ID and OOD performance in all cases, but the correlation between ID and OOD fairness was tenuous. **b**, One particular point where fairness transfers between ID and OOD datasets (‘Effusion’ with ‘age’ as the attribute; *R* = 0.98, *P* = 3.0 × 10^−^^36^, two-sided *t*-test). The center line and the shadow denote the mean and 95% CI, respectively. **c**, One particular point where fairness does not transfer between ID and OOD datasets (‘Pneumothorax’ with ‘sex and race’ as the attribute; *R* = −0.50, *P* = 4.4 × 10^−^^3^, two-sided *t*-test). The center line and the shadow denote the mean and 95% CI, respectively. **d**, The ID Pareto front for ‘Cardiomegaly’ prediction using ‘race’ as the attribute. **e**, The transformation of the ID Pareto front to the OOD Pareto front, for ‘Cardiomegaly’ prediction using ‘race’ as the attribute. Models that are Pareto optimal ID often do not maintain Pareto optimality OOD.

**Extended Data Fig. 6:**
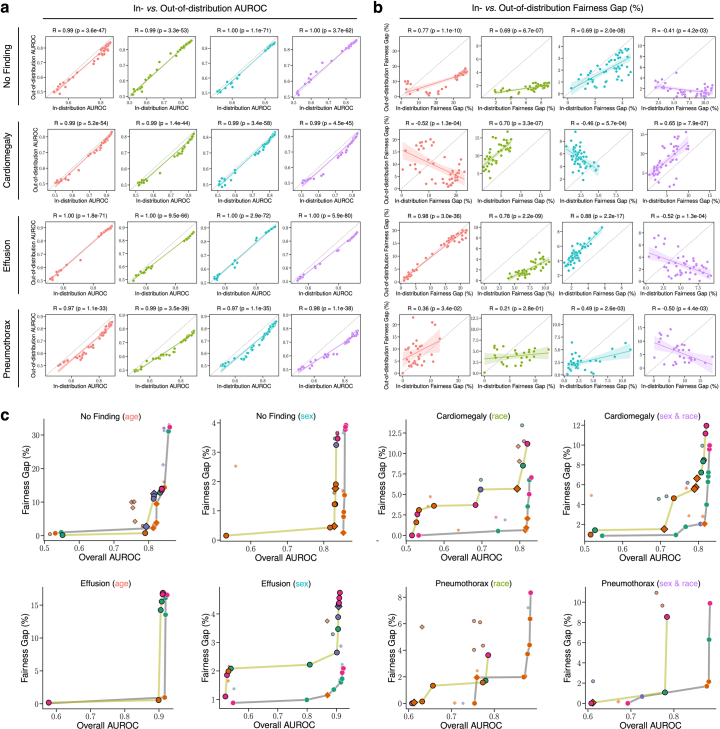
The transfer of performance (overall AUROC) and fairness between the ID (MIMIC-CXR) and OOD datasets. **a**, The OOD performance versus the ID performance for each task and attribute combination. **b**, The OOD fairness versus the ID fairness for each task and attribute combination. **c**, The Pareto front between fairness and performance for ID and OOD, finding that models that are Pareto optimal ID often do not maintain Pareto optimality OOD. All p values are calculated using two-sided t-test. The center line and the shadow denote the mean and 95% CI, respectively.

When deploying AI models in real settings, it is crucial to ensure that models can generalize to data from unseen institutions or environments. We directly tested all trained models in the OOD setting, where we report results on external test datasets that are unseen during model development. Figure 4 illustrates that the correlation between ID and OOD performance is high across different settings, which was observed in previous work^44^. However, we found that there was no consistent correlation between ID and OOD fairness. For example, Fig. 4b shows an instance where the correlation between ID fairness and OOD fairness is strongly positive (‘Effusion’ with ‘age’ as the attribute; *R* = 0.98, *P* = 3.0 × 10^−36^, two-sided *t*-test), whereas Fig. 4c shows an instance where the correlation between these metrics is actually significantly negative (‘Pneumothorax’ with ‘sex and race’ as the attribute; *R* = −0.50, *P* = 4.4 × 10^−3^, two-sided *t*-test). Across 16 combinations of task and attribute, we found that five such settings exhibited this negative correlation, and three additional settings exhibited only a weak (*R* < 0.5) positive correlation (see Extended Data Fig. 6a,b for additional correlation plots). Thus, improving ID fairness may not lead to improvements in OOD fairness, highlighting the complex interplay between fairness and distribution shift^45,46^.

In addition, we investigated whether models achieving ID Pareto optimality between fairness and performance will maintain in OOD settings. As shown for ‘Cardiomegaly’ prediction using race as the attribute, models originally on the Pareto front ID (Fig. 4d) do not guarantee to maintain Pareto optimality when deployed in a different OOD setting (Fig. 4e). We show additional examples of this phenomenon in Extended Data Fig. 6c.

### Dissecting model fairness under distribution shift

To disentangle the OOD fairness gap, we present a way to decompose model fairness under distribution shift. Specifically, we decompose and attribute the change in fairness between ID and OOD to be the difference in performance change for each of the groups—that is, the change in fairness is determined by how differently the distribution shift affects each group (Methods).

**Extended Data Fig. 7:**
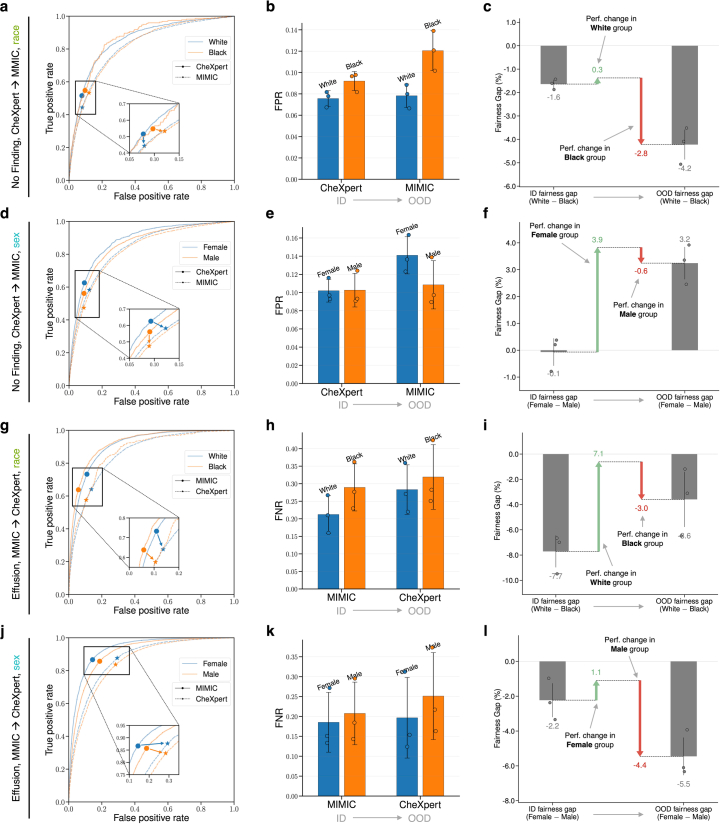
Examining the behavior of specific ERM models transferred from ID setting to OOD setting. **a, d, g, j**, The receiver operating characteristic (ROC) curves for each group and each dataset, marking the operating point of the model. **b, e, h, k**, This shift in the operating point results in a change in the FPR and FNR values of subgroups on the OOD dataset. Each bar and its error bar indicate the mean and standard deviation across 3 independent runs. **c, f, i, l**, We decompose the OOD fairness gap as a function of the ID fairness gap, and the change in FPR for each of the groups. Each bar and its error bar indicate the mean and standard deviation across 3 independent runs.

In Extended Data Fig. 7, we show examples of transferring a trained model from ID setting to OOD setting. For example, Extended Data Fig. 7d illustrates an ERM model trained to predict ‘No Finding’ on CheXpert (ID) and transferred to MIMIC-CXR (OOD) while evaluating fairness across sex. We found that the model was fair with respect to the FPR gap in the ID setting (−0.1% gap, not significant) but had a significant FPR gap when deployed in the OOD setting (3.2%), with females being underdiagnosed at a higher rate (Extended Data Fig. 7e). We then segmented this FPR gap by sex and found that females experienced an increase in FPR of 3.9%, whereas males experienced an increase in FPR of 0.8% (Extended Data Fig. 7f). In other words, the model becomes worse for both groups in an OOD setting but to a much larger extent for female patients. This decomposition suggests that mitigation strategies that reduce the impact of the distribution shift on females could be effective in reducing the OOD fairness gap in this instance.

We further extended this study to a larger set of tasks and protected attributes (Extended Data Fig. 7). Across all settings, the disparate impact of distribution shift on each group was a significant component, indicating that mitigating the impact of distribution shift is as important as mitigating ID fairness, if the goal is to achieve a fair model OOD.

### Globally optimal model selection for OOD fairness

Figure 4 shows that selecting a model based on ID fairness may not lead to a model with optimal OOD fairness. Here, we examined alternate model selection criteria that may lead to better OOD fairness, when we have access only to ID data. Our goal is to find ‘globally optimal’ models that maintain their performance and fairness in new domains. First, we subsetted our selection only to models that had satisfactory ID overall performance (defined as those with overall validation AUROC no less than 5% of the best ERM model). This set of models also had satisfactory OOD performance (Supplementary Fig. 1).

**Fig. 5:**
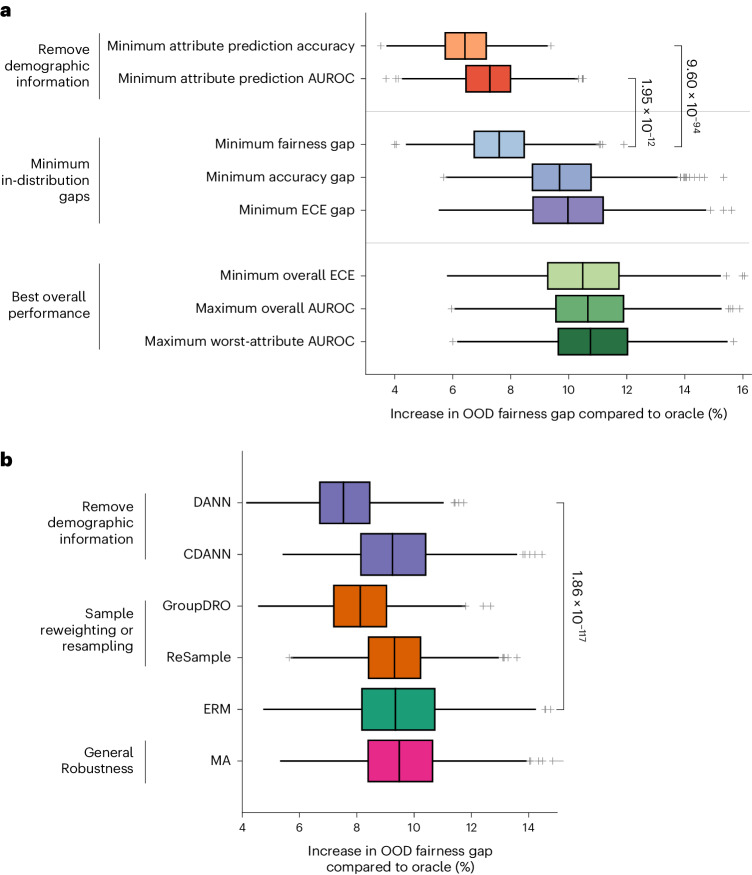
OOD fairness of models with different model selection criteria and for different algorithms. **a**, We varied the ID model selection criteria and compared the selected model against the oracle that chooses the model that is most fair OOD. We plotted the increase in OOD fairness gap of the selected model over the oracle, averaged across 42 combinations of OOD dataset, task and attribute. We used non-parametric bootstrap sampling (*n* = 1,000) to define the bootstrap distribution for the metric. We found that selection criteria based on choosing models with minimum attribute encoding achieve better OOD fairness than naively selecting based on ID fairness or other aggregate performance metrics (‘Minimum Attribute Prediction Accuracy’ versus ‘Minimum Fairness Gap’: *P* = 9.60 × 10^−94^, one-tailed Wilcoxon rank-sum test; ‘Minimum Attribute Prediction AUROC’ versus ‘Minimum Fairness Gap’: *P* = 1.95 × 10^−12^, one-tailed Wilcoxon rank-sum test). **b**, We selected the model for each algorithm with the minimum ID fairness gap. We evaluated its OOD fairness against the oracle on the same 42 settings. We found that removing demographic encoding (that is, DANN) leads to the best OOD fairness (‘DANN’ versus ‘ERM’: *P* = 1.86 × 10^−117^, one-tailed Wilcoxon rank-sum test). On each box, the central line indicates the median, and the bottom and top edges of the box indicate the 25th and 75th percentiles, respectively. The whiskers extend to 1.5 times the interquartile range. Points beyond the whiskers are plotted individually using the ‘+’ symbol.

Next, we proposed eight candidate model selection criteria (Fig. 5a), corresponding to selecting the model from this set that minimizes or maximizes some ID metric. We evaluated the selected model by its OOD fairness across five external datasets, each containing up to four attributes and up to four tasks, corresponding to a total of 42 settings. We compared the OOD fairness of the selected model to the OOD fairness of an ‘oracle’, which observes samples from the OOD dataset and directly chooses the model with the smallest OOD fairness gap. For each setting, we computed the increase in fairness gap of each selection criteria relative to the oracle. In Fig. 5a, we report the mean across the 42 settings as well as the 95% confidence interval (CI) computed from 1,000 bootstrap iterations. We found that, surprisingly, selecting the model with the minimum ID fairness gap may not be optimal. Instead, two other criteria based on selecting models where the embedding contains the least attribute information lead to a lower average OOD fairness gap. For instance, we observed a significantly lower increase in OOD fairness gap by selecting models with the ‘Minimum Attribute Prediction Accuracy’ as compared to ‘Minimum Fairness Gap’ (*P* = 9.60 × 10^−94^, one-tailed Wilcoxon rank-sum test). The result echoes our finding in Fig. 2 that the encoding of demographic attributes is positively correlated with ID fairness.

Finally, we studied the fairness of each algorithm in the OOD setting. We maintained the performance cutoff described above and selected the model for each algorithm with the lowest ID fairness gap. In Fig. 5b, we report the mean increase in OOD fairness gap relative to the oracle across the same 42 settings. We found that methods that remove demographic information from embeddings (specifically, DANN) lead to the lowest average OOD fairness gap (‘DANN’ versus ‘ERM’: *P* = 1.86 × 10^−117^, one-tailed Wilcoxon rank-sum test). Our findings demonstrate that evaluating and removing demographic information encoded by the model ID may be the key to ‘globally optimal’ models that transfer both performance and fairness to external domains.

## Discussion

We demonstrated the interplays between the demographic encoding of attributes as ‘shortcuts’ in medical imaging AI models and how they change under distribution shifts. Notably, we validated our findings across global-scale datasets in radiology (Table 1) and across multiple medical imaging modalities (Extended Data Figs. 1 and 2). The results show that algorithmic encoding of protected attributes leads to unfairness (Fig. 2) and mitigating shortcuts can reduce ID fairness gaps and maintain performance (Fig. 3). However, our results also show that there exists an inherent tradeoff for clinically meaningful metrics beyond fairness (Extended Data Fig. 5), and such fairness does not transfer under distribution shift (Fig. 4). We provide initial strategies to dissect and explain the model fairness under distribution shifts (Extended Data Fig. 7). Our results further reveal actionable algorithm and model selection strategies for OOD fairness (Fig. 5).

Our results have multiple implications. First, they offer a cautionary tale on the efficacy and consequences of eliminating demographic shortcuts in disease classification models. On the one hand, removing shortcuts addresses ID fairness, which is a crucial consideration in fair clinical decision-making^12^. On the other hand, the resulting tradeoffs with other metrics and non-transferability to OOD settings raises the question about the long-term utility in removing such shortcuts. This is particularly complex in the healthcare setting, where the relationship between demographics and the disease or outcome label is complex^47^, variables can be mislabeled^48^ and distribution shifts between domains are difficult to quantify^1^.

Second, we frame demographic features as potential ‘shortcuts’, which should not be used by the model to make disease predictions. However, some demographic variables could be a direct causal factor in some diseases (for example, sex as a causal factor of breast cancer). In these cases, it would not be desirable to remove all demographic reliance but, instead, match the reliance of the model on the demographic attribute to its true causal effect^49^. In the tasks that we examined here, demographic variables, such as race, may have an indirect effect on disease (for example, through socioeconomic status)^50^, which may vary across geographic location or even time period^51^. Whether demographic variables should serve as proxies for these causal factors is a decision that should rest with the model deployers^14,47,52,53^.

Third, we present a preliminary decomposition for diagnosing OOD model fairness changes, by expressing it as a function of the ID fairness gap and the performance change of each group. We found that the disparate impact of distribution shift on per-group performance is a major contributor to lack of fairness in OOD settings. Our work suggests that, for practitioners trying to achieve fairness in models deployed in a different domain, mitigating ID fairness is at least as important as mitigating the impact of distribution shift for particular groups. However, building models robust to arbitrary domain shifts is, in general, a challenging task^54,55^. Having some knowledge or data about how the distributions may shift, or even the ability to actively collect data for particular groups, may be necessary^56^. Developing methods and deriving theoretical characterizations of fairness under distribution shift is an active area of research^45,46^.

Fourth, the US Food & Drug Administration (FDA), as the primary regulatory body for medical technologies, does not require external validation of clinical AI models, relying instead on the assessment by the product creator^57^. Our findings underscore the necessity for regular evaluation of model performance under distribution shift^58,59^, challenging the popular opinion of a single fair model across different settings^60^. This questions the effectiveness of developer assurances on model fairness at the time of testing and highlights the need for regulatory bodies to consider real-world performance monitoring, including fairness degradation^61^. Finally, when a model is deployed in any clinical environment, both its overall and per-group performance, as well as associated clinical outcomes, should be continuously monitored^62^.

Finally, although we imply that smaller ‘fairness gaps’ are better, enforcing these group fairness definitions can lead to worse utility and performance for all groups^43,63–66^, and other fairness definitions may be better suited to the clinical setting^8,67^. We note that these invariant notions of fairness could have drawbacks^66^, as equalized odds are incompatible with calibration by group (Extended Data Fig. 5), and enforcing equalized odds often lead to the ‘leveling down’ effect in overall performance^63,64^. We present the Pareto curve showing the tradeoff between fairness and accuracy, allowing the practitioner to select a model that best fits their deployment scenario. In general, we encourage practitioners to choose a fairness definition that is best suited to their use case and carefully consider the performance–equality tradeoff. The impact of minimizing algorithmic bias on real-world health disparities, the ultimate objective, is complex^68^, and there is no guarantee that deploying a fair model will lead to equitable outcomes. In addition, although we constructed several models for clinical risk prediction, we do not advocate for deployment of these models in real-world clinical settings without practitioners carefully testing models on their data and taking other considerations into account (for example, privacy, regulation and interpretability)^1,3^.

## Methods

### Datasets and pre-processing

The datasets used in this study are summarized in Extended Data Table 1. Unless otherwise stated, we trained models on MIMIC-CXR^21^ and evaluated on an OOD dataset created by merging CheXpert^22^, NIH^23^, SIIM^24^, PadChest^25^ and VinDr^26^. We included all images (both frontal and lateral) and split each dataset into 70% train, 15% validation and 15% test sets. Note that only MIMIC-CXR and CheXpert have patient race information available, and we extracted race (and other attributes) following established protocols^69^. For MIMIC-CXR, demographic information was obtained by merging with MIMIC-IV^70^. For CheXpert, separate race labels were obtained from the Stanford Center for Artificial Intelligence in Medicine & Imaging (https://aimi.stanford.edu/) website. Where applicable, we dropped patients with missing values for any attribute.

For all datasets, we excluded samples where the corresponding patient has missing age or sex. For ODIR and ISIC, we dropped samples from patients younger than 18 years and older than 80 years due to small sample sizes (that is, smaller than 3% of the total dataset).

Owing to computational constraints, we mainly chose four prediction tasks for CXRs (that is, ‘No Finding’, ‘Effusion’, ‘Cardiomegaly’ and ‘Pneumothorax’). We selected these tasks for several reasons: (1) diversity in presentation: ‘Effusion’, ‘Cardiomegaly’ and ‘Pneumothorax’ each present distinctively and occur in different locations on a CXR, allowing for a comprehensive evaluation across varied pathologies and underlying causes; (2) prevalence in clinical and research settings: these labels are not only common in clinical practice but also frequently studied in prior academic work^7,12,63^ and used in commercial diagnostic systems^71^; and (3) performance and fairness considerations: these labels are among those with both the highest diagnostic accuracy and substantial fairness gaps on MIMIC-CXR, making them particularly relevant for exploring the relationship between model performance and fairness^7,12^.

We scaled all images to 224 × 224 for input to the model. We applied the following image augmentations during training only: random flipping of the images along the horizontal axis, random rotation of up to 10° and a crop of a random size (70–100%) and a random aspect ratio (3/4 to 4/3).

### Evaluation methods

To evaluate the performance of disease classification in medical imaging, we used the following metrics: AUROC, TPR, TNR and ECE.

The TPR and TNR are calculated as (FN, false negative; FP, false positive; TP, true positive; TN, true negative):

$${\mathrm{TPR}}=\frac{{\mathrm{TP}}}{{\mathrm{TP}}+{\mathrm{FN}}}$$

$${\mathrm{TNR}}=\frac{{\mathrm{TN}}}{{\mathrm{TN}}+{\mathrm{FP}}}$$

When reporting the sensitivity and specificity, we followed previous work^12,72^ in selecting the threshold that maximizes the F1 score. This threshold optimization procedure is conducted separately for each dataset, task, algorithm and attribute combination. We followed standard procedures to calculate the 95% CI for sensitivity and specificity.

We also reported AUC, which is the area under the corresponding ROC curves showing an aggregate measure of detection performance. Finally, we report the expected calibration error (ECE)^73^, which we computed using the netcal library^74^.

#### Assessing the fairness of machine learning models

To assess the fairness of machine learning models, we evaluated the metrics described above for each demographic group as well as the difference in the value of the metric between groups. Equality of TPR and TNR between demographic groups is known in the algorithmic fairness literature as equal odds^75^. As the models that we studied in this work are likely to be used as screening or triage tools, the cost of an FP may be different from the cost of an FN. In particular, for ‘No Finding’ prediction, FPs (corresponding to underdiagnosis^12^) would be more costly than FNs, and so we focused on the FPR (or TNR) for this task. For all remaining disease prediction tasks, we focused on the FNR (or TPR) for the same reason. Equality in one of the class-conditioned error rates is an instance of equal opportunity^30^.

Finally, we also examined the per-group ECE and ECE gap between groups. Note that zero ECE for both groups (that is, calibration per group) implies the fairness definition known as sufficiency of the risk score^75^. We emphasize that differences in calibration between groups is a significant source of disparity, as consistent under-estimation or over-estimation of risk for a particular group could lead to under-treatment or over-treatment for that group at a fixed operating threshold relative to the true risk^76^.

### Quantifying the distribution shifts

We examined and quantified the types and degrees of distribution shifts in both ID and OOD settings in this study. Inspired by previous work^46,77^, we performed a series of hypothesis tests to determine if there were significant statistical differences in distributions between demographic groups and across different pairs of datasets. All *P* values were adjusted for multiple testing using Bonferroni correction^78^.

#### ID setting

We studied the following distribution shifts in the ID setting:

##### Prevalence shift: P(Y|A)

For binary outcomes Y across groups, we calculated the total variational distance between the probability distributions of Y conditioned on different groups and used a two-sample binomial proportion test, where the null hypothesis corresponds to P(Y|A = *a*_1_) = P(Y|A = *a*_2_):

$${d}_{\mathrm{Y}}({a}_{1},{a}_{2})={d}_{\mathrm{TV}}({\mathrm{P}}({\mathrm{Y}}|{\mathrm{A}}={a}_{1}),{\mathrm{P}}({\mathrm{Y}}|{\mathrm{A}}={a}_{2}))$$

##### Representation shift: P(X|A)

When comparing the distribution of the input images X, we first encoded them into representations derived from a frozen foundation model *f* that is trained in a self-supervised manner on diverse CXR datasets^79,80^. We then used the mean maximum discrepancy (MMD) distance and a permutation-based hypothesis test following ref. ^81^ to test if demographic groups differed statistically in their distribution of representations:

$$\left.{d}_{\mathrm{X}}({a}_{1},{a}_{2})={d}_{\mathrm{MMD}}\left({\mathrm{P}}\left(\;f({\mathrm{X}})\right|{\mathrm{A}}={a}_{1}\right),{\mathrm{P}}\left(f({\mathrm{X}})|{\mathrm{A}}={a}_{2}\right)\right)$$

#### OOD setting

We studied the following distribution shifts in the OOD setting (the null hypothesis is P_ID_(·) = P_OOD_(·)):

##### Label shift: P(Y)

We calculated the total variational distance between the probability distributions of binary outcomes Y across ID and OOD datasets using a two-sample binomial proportion test:

$${d}_{\mathrm{Y}}={d}_{\mathrm{TV}}({\mathrm{P}}_{\mathrm{ID}}\left({\mathrm{Y}}\right),{\mathrm{P}}_{\mathrm{OOD}}({\mathrm{Y}}))$$

##### Prevalence shift: P(Y|A = a)

We similarly evaluated the distance between the distributions of Y conditioned on specific demographic subgroups (A) between ID and OOD datasets:

$${d}_{{\mathrm{Y}}{\rm{|}}{\mathrm{A}}}(a)={d}_{\mathrm{TV}}({\mathrm{P}}_{\mathrm{ID}}\left({\mathrm{Y}}{\rm{|}}{\mathrm{A}}=a\right),{\mathrm{P}}_{\mathrm{OOD}}({\mathrm{Y}}{\rm{|}}{\mathrm{A}}=a))$$

##### Covariate shift: P(X)

We again encoded X into representations derived from a frozen foundation model *f* and then used the MMD distance and a permutation-based hypothesis test^81^ to examine if ID and OOD datasets differed statistically in their distribution of representations:

$${d}_{\mathrm{X}}={d}_{\mathrm{MMD}}({\mathrm{P}}_{\mathrm{ID}}\left(f({\mathrm{X}})\right),{\mathrm{P}}_{\mathrm{OOD}}(\;f({\mathrm{X}}\,)))$$

##### Representation shift: P(X|A = a)

Similarly, we calculated the MMD distance conditioned on subgroup A to evaluate shifts in the representation space:

$${d}_{{\mathrm{X}}{{|}}A}(a)={d}_{\mathrm{MMD}}({\mathrm{P}}_{\mathrm{ID}}\left(\;f\left({\mathrm{X}}\right){{|}}{\mathrm{A}}=a\right),{\mathrm{P}}_{\mathrm{OOD}}\left(\;f\left({\mathrm{X}}\right)|{\mathrm{A}}=a\right))$$

We provide additional results on quantifying various distribution shifts in both ID and OOD settings in the Supplementary Information (Supplementary Tables 1–3).

### Training details

We trained DenseNet-121 (ref. ^33^) models on each task, initializing with ImageNet^82^ pre-trained weights. We evaluated six algorithms: ERM^34^, ReSample^35^, GroupDRO^36^, DANN^37^, CDANN^38^ and MA^39^.

For each combination of task, algorithm and demographic attribute, we conducted a random hyperparameter search^83^ with 12 runs. During training, for a particular attribute, we evaluated the validation set worst-group validation AUROC every 1,000 steps and early stopped if this metric had not improved for five evaluations. We tuned the learning rate and weight decay for all algorithms and also tuned algorithm-specific hyperparameters as mentioned in the original works. We selected the hyperparameter setting that maximized the worst-attribute validation AUROC. CIs were computed as the standard deviation across three different random seeds for each hyperparameter setting.

We also explored a multi-label training setup, where models were trained simultaneously on 14 binary labels available in MIMIC-CXR^7^. We followed the same experimental protocol as outlined in the main paper, including hyperparameter tuning and model selection. Our findings in the multi-label setup mirrored those seen in the binary task setup (Supplementary Figs. 2 and 3).

To obtain the level of demographic encoding within representations (Fig. 2), we first computed representations using a trained disease prediction model. We froze these representations and trained a multi-class multi-nomial logistic regression model to predict the demographic group using the training set using the scikit-learn library^84^. We varied the L2 regularization strength between 10^−5^ and 10 and selected the model with the best macro-averaged AUROC on the validation set. We report the macro-averaged AUROC on the test set.

### Decomposing OOD fairness

Here we present a first approach toward decomposing the fairness gap in an OOD environment as a function of the ID fairness gap and the impact that the distribution shift has in each group. In particular, let *D*_src_ and *D*_tar_ be the source and target datasets, respectively. Let *g ∈ G* be a particular group from a set of groups. Let *L*_*f*_(*g*, *D*) be an evaluation metric for a model *f*, which is decomposable over individual samples—that is, *L*_*f*_(*g*, *D*) = ${L}_{f}(g,D)={\sum }_{(x,y,g{\prime} )\in D;g{\prime} =g}l(f(x),y)$. Examples of such metrics are the accuracy, TPR or TNR. Then, we can decompose:

$$\begin{array}{c}{L}_{f}\,({g}_{1},{D}_{\mathrm{tar}})-{L}_{f}\,({g}_{2},{D}_{\mathrm{tar}})=[{L}_{f}\,({g}_{1},{D}_{\mathrm{src}})-{L}_{f}\,({g}_{2},{D}_{\mathrm{src}})]\\ +[{L}_{f}\,({g}_{2},{D}_{\mathrm{src}})-{L}_{f}\,({g}_{2},{D}_{\mathrm{tar}})]-[{L}_{f}\,({g}_{1},{D}_{\mathrm{src}})-{L}_{f}\,({g}_{1},{D}_{\mathrm{tar}})].\end{array}$$

The left-hand term is the fairness gap in the OOD environment, and the three terms on the right are (1) the fairness gap in the ID data, (2) the impact of the distribution shift on g_2_ and (3) the impact of the distribution shift on g_1_. We note that, to achieve a low fairness gap in the OOD environment, it is important not only to minimize the ID fairness gap (term 1) but also to minimize the difference in how the distribution shift impacts each group (term 2 − term 3).

### Evaluation with different medical imaging modalities

In addition to radiology, we also examined medical AI applications in dermatology and ophthalmology to corroborate our findings. Specifically, Extended Data Fig. 1 shows the results for dermatological imaging. We used the ISIC dataset^28^, which contains 32,259 images sourced from multiple international sites. We focused on the ‘No Finding’ task, taking into account ‘sex’ and ‘age’ as the sensitive demographic attributes (Extended Data Fig. 1a). Similar to our observations in radiology, we identified fairness gaps within subgroups based on age and sex (Extended Data Fig. 1b), although these disparities were less significant than those observed in CXR assessments (for example, fairness gaps smaller than 2%). This was further confirmed by the Pareto front plot, where most models, including ERM, could achieve a good performance–fairness tradeoff (Extended Data Fig. 1c).

We extended our analysis to ophthalmology images, specifically focusing on retinopathy detection, using the ODIR dataset^29^ with 6,800 images (Extended Data Fig. 2). The task that we considered was ‘Retinopathy’, with ‘sex’ and ‘age’ being used as demographic attributes (Extended Data Fig. 2a). Notably, significant subgroup fairness gaps were observed in age (43% FNR gap between groups ‘60–80’ and ‘18–40’). In contrast, the fairness gap based on sex was less significant, with a 3% FNR difference between ‘female’ and ‘male’ subgroups. We further verified the Pareto front for both attributes, where similar observations hold that algorithms for fixing demographic shortcuts could improve ID fairness while incurring minimal detriments to performance (measured in AUROC).

### Analysis on underdiagnosis versus overdiagnosis

In evaluating fairness metrics, our primary study centered on underdiagnosis, specifically the disparities in FPR for ‘No Finding’ and discrepancies in FNR for other conditions. However, an alternative approach involves focusing on overdiagnosis, defined as variances in FNR for ‘No Finding’ and differences in FPR for other diseases. We present findings between their relationship in Extended Data Fig. 3. An analysis spanning two datasets (MIMIC and CheXpert) and various tasks revealed a consistent pattern: larger gaps in underdiagnosis tend to correspond with more significant overdiagnosis discrepancies. Nonetheless, certain task and attribute combinations exhibited more complex trends, indicating a necessity for deeper exploration and informed decision-making regarding the most appropriate fairness metrics for critical disease evaluations in practical medical settings.

### Direct prediction of demographic attributes

We provide analyses for demographic encoding of attribute information. In the main paper, we analyzed the predictiveness of attributes (for example, age, sex and race) based on the embeddings from a disease classification model. The distinct predictiveness between attributes in these domains could be attributed to the intrinsic characteristics of the datasets or the nature of the conditions being studied. To delve deeper, we conducted an experiment training an ERM model to predict these attributes directly using the dermatology dataset (ISIC), and we show the results in Supplementary Table 4. We observed that certain attributes are indeed less predictive compared to others (that is, age versus sex), suggesting that age may be inherently more challenging to encode within the studied dermatology dataset. Furthermore, the results reveal variations in the predictiveness of age across different subgroups (for example, age groups ‘18–40’ and ‘60–80’ exhibit higher AUROC than the ‘40–60’ group).

### Analysis using multi-label models

Prior works^7^ studied CXR classification in the multi-label setting, where a model contains an encoder, followed by an individual linear classification head for each of the downstream tasks. We followed the setup^7^ to study the following 14 binary labels in MIMIC-CXR: Atelectasis, Cardiomegaly, Consolidation, Edema, Enlarged Cardiomediastinum, Fracture, Lung Lesion, Airspace Opacity, No Finding, Effusion, Pleural Other, Pneumonia, Pneumothorax and Support Devices.

We adapted the following methods to the multi-label setting, (1) ERM, (2) DANN and (3) CDANN, and we followed the same experimental protocol in the main paper in terms of hyperparameter tuning. Note that GroupDRO and ReSample are challenging to adapt to the multi-label setting, as the number of groups is exponential in the number of tasks. For each combination of algorithm and attribute, we selected the multi-label model that maximizes the worst-attribute AUROC, averaged across the 14 tasks.

First, we examined the level of demographic encoding present in the embeddings of the best multi-task ERM model and found that it also encodes a variety of demographic information, similar to the single-label case (Supplementary Fig. 2a). We further showed the fairness gaps of this best multi-label ERM model and observed that a variety of fairness gaps exist and are statistically significant across all tasks (Supplementary Fig. 2b). In addition, we plotted the correlation between the fairness gap and the attribute prediction AUROC, across all trained multi-label models. We found a strong and statistically significant positive correlation among all combinations of task and attribute, similar to the single-label case (Supplementary Fig. 2c).

We also present Pareto plots showing the tradeoff between the fairness gap and overall AUROC across all models, for each combination of task and attribute (Supplementary Fig. 3). Overall, we found that the Pareto fronts for the multi-label models demonstrate similar behavior as the single-task models—where the multi-label ERM exhibits the best overall AUROC but has high fairness gap. In addition, with debiasing methods such as multi-label DANN and CDANN, we are able to achieve fair models with minimal loss in overall AUROC.

### Test set rebalancing for prevalence shift

We investigated whether eliminating the prevalence shift in the test set would address the observed fairness gaps. Following prior work^40^, we balanced the test set for multiple attributes—age and race—ensuring that demographic proportions (for example, ‘White’ aged ‘20–40’ versus ‘Black’ aged ‘60–80’) and disease prevalence are uniform across all attribute combinations. This approach aims to eliminate prevalence shifts within the test set. Our findings in Supplementary Fig. 4 suggest that, although test set rebalancing can reduce fairness gaps for certain task and attribute combinations (for example, ‘No Finding’ for ‘race’ and ‘Cardiomegaly’ for ‘age’, as compared to Fig. 2b), there exists significant gaps even after rebalancing the test set, indicating that fairness gaps are influenced by multiple shifts beyond just prevalence shifts.

### Statistical analysis

#### Correlation

To calculate the correlations between variables, we used Pearson correlation coefficients and their associated *P* value (two-sided *t*-test, α = 0.05). 95% CI for the Pearson correlation coefficient was calculated.

#### Increase in OOD fairness gap

One-tailed Wilcoxon rank-sum test (α = 0.05) was used to assess the increase in OOD fairness gap compared to oracle models.

#### CIs

We used non-parametric bootstrap sampling to generate CIs: random samples of size *n* (equal to the size of the original dataset) were repeatedly sampled 1,000 times from the original dataset with replacement. We then estimated the increase in OOD fairness gap compared to oracle using each bootstrap sample (α = 0.05).

All statistical analysis was performed with Python version 3.9 (Python Software Foundation).

### Reporting summary

Further information on research design is available in the Nature Portfolio Reporting Summary linked to this article.

## Online content

Any methods, additional references, Nature Portfolio reporting summaries, source data, extended data, supplementary information, acknowledgements, peer review information; details of author contributions and competing interests; and statements of data and code availability are available at 10.1038/s41591-024-03113-4.

## Supplementary information

Supplementary Tables 1–4, Figs. 1–4 and Note 1.

Reporting Summary

## Figures and Tables

**Extended Data Table 1:**
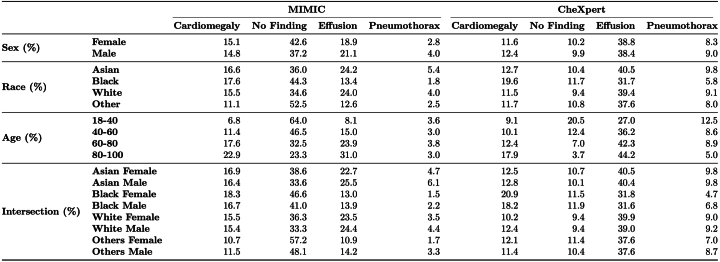
Prevalence rates for each demographic subgroup of the datasets used for training models in this study

**Extended Data Table 2:**
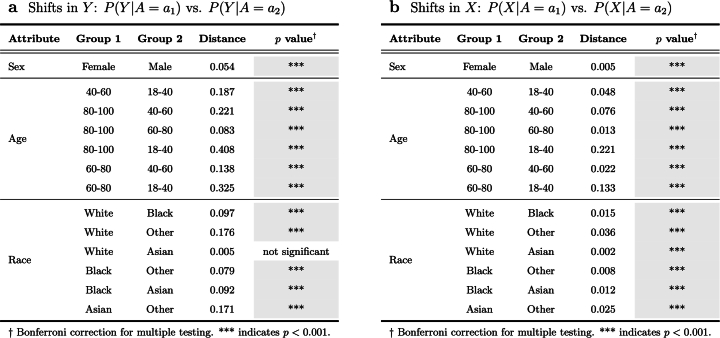
Distribution shift quantification in the ID settings between demographic groups on MIMIC-CXR

**a**, Prevalence shift P(Y|A) was derived using the total variational distance between the probability distributions of Y conditioned on different groups. *P* values were computed using a two-sided proportion *z*-test. **b**, Representation shift P(X|A) was derived by first encoding input into representations from a frozen foundation model *f* (that is, MedCLIP^79^) and then computing the MMD distance with a Gaussian kernel^81^. *P* values were computed using a two-sided permutation test using this distance as the test statistic^81^. All *P* values were adjusted for multiple testing using Bonferroni correction^78^.

**Extended Data Table 3:**
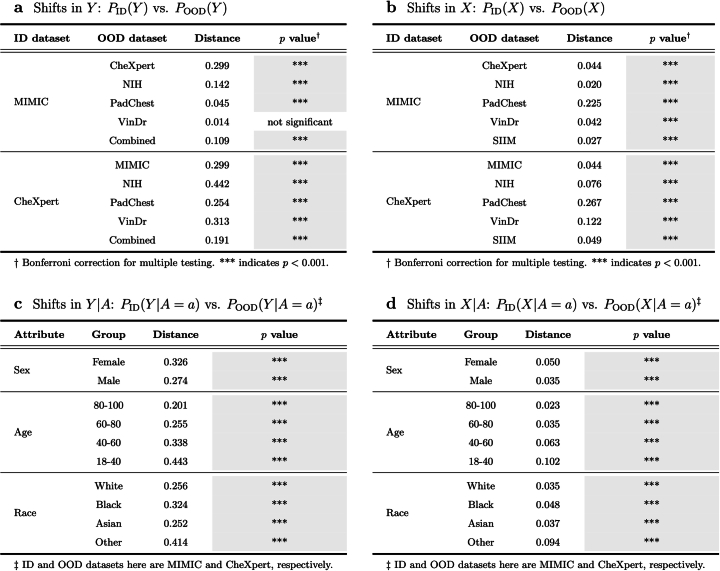
Distribution shift quantification in the OOD settings between different datasets

**a**, Label shift P(Y) was derived using the total variational distance between the probability distributions of Y between ID and OOD datasets. *P* values were computed using a two-sided proportion *z*-test. **b**, Covariate shift P(X) was derived by first encoding input into representations from a frozen foundation model *f* (that is, MedCLIP^79^) and then computing the MMD distance with a Gaussian kernel^81^ between ID and OOD datasets. *P* values were computed using a two-sided permutation test using this distance as the test statistic^81^. **c**, Prevalence shift P(Y|A = a) was derived using the total variational distance conditioned on specific subgroups between ID and OOD datasets. *P* values were computed using a two-sided proportion *z*-test. **d**, Representation shift P(X|A = a) was derived using the MMD distance on specific subgroups between ID and OOD datasets^81^. *P* values were computed using a two-sided permutation test using this distance as the test statistic^81^. All *P* values were adjusted for multiple testing using Bonferroni correction^78^.

## Data Availability

All datasets used in this study are publicly available. The MIMIC-CXR (https://www.physionet.org/content/mimic-cxr-jpg/2.1.0/) and VinDr-CXR (https://physionet.org/content/vindr-cxr/1.0.0/) datasets are available from PhysioNet after completion of a data use agreement and a credentialing procedure. The CheXpert dataset (https://stanfordmlgroup.github.io/competitions/chexpert/), along with associated race labels, is available from the Stanford Center for Artificial Intelligence in Medicine & Imaging website (https://aimi.stanford.edu/). The ChestX-ray14 (National Institutes of Health) dataset (https://nihcc.app.box.com/v/ChestXray-NIHCC) is available to download from the National Institutes of Health Clinical Center. The PadChest dataset (https://academictorrents.com/details/96ebb4f92b85929eadfb16761f310a6d04105797) can be downloaded from the Medical Imaging Databank of the Valencia Region. The SIIM-ACR Pneumothorax Segmentation dataset (https://www.kaggle.com/c/siim-acr-pneumothorax-segmentation) can be downloaded from its Kaggle contest page. The ISIC 2020 dataset (https://challenge.isic-archive.com/data/#2020) can be downloaded from the SIIM-ISIC Melanoma Classification Challenge page. The ODIR dataset (https://odir2019.grand-challenge.org/dataset/) can be obtained from the ODIR 2019 challenge hosted by Grand Challenges.

## Code availability

Code that supports the findings of this study is publicly available with an open-source license at https://github.com/YyzHarry/shortcut-ood-fairness.

## References

[1] Zhang, A., Xing, L., Zou, J. & Wu, J. C. Shifting machine learning for healthcare from development to deployment and from models to data. *Nat. Biomed. Eng.* **6**, 1330–1345 (2022). 35788685 10.1038/s41551-022-00898-y PMC12063568

[2] Sendak, M. P. et al. A path for translation of machine learning products into healthcare delivery. *EMJ Innov*. 10.33590/emjinnov/19-00172 (2020).

[3] Wiens, J. et al. Do no harm: a roadmap for responsible machine learning for health care. *Nat. Med.* **25**, 1337–1340 (2019). 31427808 10.1038/s41591-019-0548-6

[4] Ahmad, M. A., Patel, A., Eckert, C., Kumar, V. & Teredesai, A. Fairness in machine learning for healthcare. In *Proc. 26th ACM SIGKDD International Conference on Knowledge Discovery & Data Mining* 3529–3530 (Association for Computing Machinery, 2020).

[5] McKinney, S. M. et al. International evaluation of an AI system for breast cancer screening. *Nature* **577**, 89–94 (2020). 31894144 10.1038/s41586-019-1799-6

[6] Burlina, P. et al. Utility of deep learning methods for referability classification of age-related macular degeneration. *JAMA Ophthalmol.* **136**, 1305–1307 (2018). 30193354 10.1001/jamaophthalmol.2018.3799 PMC6248178

[7] Seyyed-Kalantari, L., Liu, G., McDermott, M., Chen, I. Y. & Ghassemi, M. *CheXclusion: Fairness Gaps in Deep Chest X-ray Classifiers*. Pacific Symposium on Biocomputing (World Scientific Publishing Company, 2020); https://psb.stanford.edu/psb-online/proceedings/psb21/seyyed-kalantari.pdf 33691020

[8] Zong, Y., Yang, Y. & Hospedales, T. MEDFAIR: benchmarking fairness for medical imaging. In *Proc. 11th International Conference on Learning Representations* (ICLR, 2023); https://openreview.net/forum?id=6ve2CkeQe5S

[9] Kinyanjui, N. M. et al. Fairness of classifiers across skin tones in dermatology. In *Medical Image Computing and Computer Assisted Intervention**—**MICCAI 2020* Vol. 12266 (eds Martel, A. L. et al.) 320–329 (Springer, 2020); 10.1007/978-3-030-59725-2_31

[10] Lin, M. et al. Improving model fairness in image-based computer-aided diagnosis. *Nat. Commun.* **14**, 6261 (2023). 37803009 10.1038/s41467-023-41974-4 PMC10558498

[11] Weng, N., Bigdeli, S., Petersen, E. & Feragen, A. Are sex-based physiological differences the cause of gender bias for chest X-ray diagnosis? In *Clinical Image-Based Procedures, Fairness of AI in Medical Imaging, and Ethical and Philosophical Issues in Medical Imaging (CLIP 2023, EPIMI 2023, FAIMI 2023)* Vol. 14242 (eds Wesarg, S. et al.) 142–152 (Springer, 2023); 10.1007/978-3-031-45249-9_14

[12] Seyyed-Kalantari, L., Zhang, H., McDermott, M. B., Chen, I. Y. & Ghassemi, M. Underdiagnosis bias of artificial intelligence algorithms applied to chest radiographs in under-served patient populations. *Nat. Med.* **27**, 2176–2182 (2021). 34893776 10.1038/s41591-021-01595-0 PMC8674135

[13] Adamson, A. S. & Smith, A. Machine learning and health care disparities in dermatology. *JAMA Dermatol.* **154**, 1247–1248 (2018). 30073260 10.1001/jamadermatol.2018.2348

[14] McCradden, M. D., Joshi, S., Mazwi, M. & Anderson, J. A. Ethical limitations of algorithmic fairness solutions in health care machine learning. *Lancet Digit. Health* **2**, e221–e223 (2020). 33328054 10.1016/S2589-7500(20)30065-0

[15] Gichoya, J. W. et al. AI recognition of patient race in medical imaging: a modelling study. *Lancet Digit. Health* **4**, e406–e414 (2022). 35568690 10.1016/S2589-7500(22)00063-2 PMC9650160

[16] Adleberg, J. et al. Predicting patient demographics from chest radiographs with deep learning. *J. Am. Coll. Radiol.* **19**, 1151–1161 (2022). 35964688 10.1016/j.jacr.2022.06.008

[17] Geirhos, R. et al. Shortcut learning in deep neural networks. *Nat. Mach. Intell.* **2**, 665–673 (2020).

[18] Banerjee, I. et al. ‘Shortcuts’ causing bias in radiology artificial intelligence: causes, evaluation, and mitigation. *J. Am. Coll. Radiol.* **20**, 842–851 (2023). 37506964 10.1016/j.jacr.2023.06.025 PMC11192466

[19] Zech, J. R. et al. Variable generalization performance of a deep learning model to detect pneumonia in chest radiographs: a cross-sectional study. *PLoS Med.* **15**, e1002683 (2018). 30399157 10.1371/journal.pmed.1002683 PMC6219764

[20] DeGrave, A. J., Janizek, J. D. & Lee, S.-I. AI for radiographic COVID-19 detection selects shortcuts over signal. *Nat. Mach. Intell.* **3**, 610–619 (2021).

[21] Johnson, A. E. et al. MIMIC-CXR-JPG, a large publicly available database of labeled chest radiographs. Preprint at https://arxiv.org/abs/1901.07042 (2019). 10.1038/s41597-019-0322-0 PMC6908718 31831740

[22] Irvin, J. et al. CheXpert: a large chest radiograph dataset with uncertainty labels and expert comparison. In *Proc. of the AAAI Conference on Artificial Intelligence* Vol. 33, 590–597 (Association for Computing Machinery, 2019); 10.1609/aaai.v33i01.3301590

[23] Wang, X. et al. ChestX-ray8: hospital-scale chest x-ray database and benchmarks on weakly-supervised classification and localization of common thorax diseases. In *Proc. of the IEEE Conference on Computer Vision and Pattern Recognition* 2097–2106 (IEEE, 2017); https://openaccess.thecvf.com/content_cvpr_2017/papers/Wang_ChestX-ray8_Hospital-Scale_Chest_CVPR_2017_paper.pdf

[24] Zawacki, A. et al. SIIM-ACR pneumothorax segmentation. *kaggle* https://www.kaggle.com/c/siim-acr-pneumothorax-segmentation/ (2019).

[25] Bustos, A., Pertusa, A., Salinas, J.-M. & De La Iglesia-Vaya, M. PadChest: a large chest x-ray image dataset with multi-label annotated reports. *Med. Image Anal.* **66**, 101797 (2020). 32877839 10.1016/j.media.2020.101797

[26] Nguyen, H. Q. et al. VinDr-CXR: an open dataset of chest X-rays with radiologist’s annotations. *Sci. Data* **9**, 429 (2022). 35858929 10.1038/s41597-022-01498-w PMC9300612

[27] Larrazabal, A. J., Nieto, N., Peterson, V., Milone, D. H. & Ferrante, E. Gender imbalance in medical imaging datasets produces biased classifiers for computer-aided diagnosis. *Proc. Natl Acad. Sci. USA* **117**, 12592–12594 (2020). 32457147 10.1073/pnas.1919012117 PMC7293650

[28] Rotemberg, V. et al. A patient-centric dataset of images and metadata for identifying melanomas using clinical context. *Sci. Data* **8**, 34 (2021). 33510154 10.1038/s41597-021-00815-z PMC7843971

[29] Ocular disease recognition. *kaggle* https://www.kaggle.com/datasets/andrewmvd/ocular-disease-recognition-odir5k (accessed 5 September 2023).

[30] Hardt, M., Price, E. & Srebro, N. Equality of opportunity in supervised learning. In *Proc. 30th Conference on Neural Information Processing Systems* (NIPS, 2016); https://proceedings.neurips.cc/paper_files/paper/2016/file/9d2682367c3935defcb1f9e247a97c0d-Paper.pdf

[31] Brown, A. et al. Detecting shortcut learning for fair medical AI using shortcut testing. *Nat. Commun.* **14**, 4314 (2023). 37463884 10.1038/s41467-023-39902-7 PMC10354021

[32] Makar, M. et al*.* Causally motivated shortcut removal using auxiliary labels. In *Proc. 25th International Conference on Artificial Intelligence and Statistics* Vol. 151, 739–766 (PMLR, 2022); https://proceedings.mlr.press/v151/makar22a/makar22a.pdf

[33] Huang, G., Liu, Z., Van Der Maaten, L. & Weinberger, K. Q. Densely connected convolutional networks. In *Proc. of the 2017 IEEE Conference on Computer Vision and Pattern Recognition* 4700–4708 (IEEE, 2017); https://doi.ieeecomputersociety.org/10.1109/CVPR.2017.243

[34] Vapnik, V. Principles of risk minimization for learning theory. In *Advances in Neural Information Processing Systems 4* (NeurIPS, 1991); https://proceedings.neurips.cc/paper_files/paper/1991/file/ff4d5fbbafdf976cfdc032e3bde78de5-Paper.pdf

[35] Idrissi, B. Y., Arjovsky, M., Pezeshki, M. & Lopez-Paz, D. Simple data balancing achieves competitive worst-group-accuracy. In *Proc. 1st Conference on Causal Learning and Reasoning* 336–351 (PMLR, 2022); https://proceedings.mlr.press/v177/idrissi22a.html

[36] Sagawa, S., Koh, P. W., Hashimoto, T. B. & Liang, P. Distributionally robust neural networks for group shifts: on the importance of regularization for worst-case generalization. In *Proc. of the International Conference on Learning Representations* (ICLR, 2020); https://openreview.net/pdf?id=ryxGuJrFvS

[37] Ganin, Y. et al. Domain-adversarial training of neural networks. *J. Mach. Learn. Res.* **17**, 2096–2030 (2016).

[38] Li, Y. et al. Deep domain generalization via conditional invariant adversarial networks. In *Proc. of the European Conference on Computer Vision (ECCV)* 624–639 (ECCV, 2018); https://openaccess.thecvf.com/content_ECCV_2018/papers/Ya_Li_Deep_Domain_Generalization_ECCV_2018_paper.pdf

[39] Polyak, B. T. & Juditsky, A. B. Acceleration of stochastic approximation by averaging. *SIAM J. Control Optim.* **30**, 838–855 (1992).

[40] Glocker, B., Jones, C., Bernhardt, M. & Winzeck, S. Algorithmic encoding of protected characteristics in chest X-ray disease detection models. *EBioMedicine* **89**, 104467 (2023). 10.1016/j.ebiom.2023.104467 PMC10025760 36791660

[41] Jones, C., Roschewitz, M. & Glocker, B. The role of subgroup separability in group-fair medical image classification. In *Medical Image Computing and Computer Assisted Intervention**—**MICCAI 2023* Vol. 14222 (eds Greenspan, H. et al.) 179–188 (Springer, 2023); 10.1007/978-3-031-43898-1_18

[42] Wei, S. & Niethammer, M. The fairness-accuracy Pareto front. *Stat. Anal. Data Min.* **15**, 287–302 (2022).

[43] Kleinberg, J., Mullainathan, S. & Raghavan, M. Inherent tradeoffs in the fair determination of risk scores. In *Proc. 8th Innovations in Theoretical Computer Science Conference (ITCS 2017)* Vol. 67 (ed. Papadimitriou, C. H.) 1–23 (2017); 10.4230/LIPIcs.ITCS.2017

[44] Miller, J. P. et al. Accuracy on the line: on the strong correlation between out-of-distribution and in-distribution generalization. In *Proc. 38th International Conference on Machine Learning* 7721–7735 (PMLR, 2021); https://proceedings.mlr.press/v139/miller21b/miller21b.pdf

[45] An, B., Che, Z., Ding, M. & Huang, F. Transferring fairness under distribution shifts via fair consistency regularization. In *Proc. 36th Conference on Neural Information Processing Systems* (NeurIPS, 2022); https://proceedings.neurips.cc/paper_files/paper/2022/file/d1dbaabf454a479ca86309e66592c7f6-Paper-Conference.pdf

[46] Schrouff, J. et al. Diagnosing failures of fairness transfer across distribution shift in real-world medical settings. In *Proc. 36th Conference on Neural Information Processing Systems* (NeurIPS, 2022); https://proceedings.neurips.cc/paper_files/paper/2022/file/7a969c30dc7e74d4e891c8ffb217cf79-Paper-Conference.pdf

[47] Vyas, D. A., Eisenstein, L. G. & Jones, D. S. Hidden in plain sight—reconsidering the use of race correction in clinical algorithms. *N. Engl. J. Med.* **383**, 874–882 (2020). 32853499 10.1056/NEJMms2004740

[48] Jain, S. et al. VisualCheXbert: addressing the discrepancy between radiology report labels and image labels. In *Proc. of the Conference on Health, Inference, and Learning* 105–115 (Association for Computing Machinery, 2021); 10.1145/3450439.3451862

[49] Kumar, A., Deshpande, A. & Sharma, A. Causal effect regularization: automated detection and removal of spurious attributes. In *Proc. 37th Conference on Neural Information Processing Systems* https://openreview.net/pdf?id=V5Oh7Aqfft (NeurIPS, 2023).

[50] Basu, A. Use of race in clinical algorithms. *Sci. Adv.* **9**, eadd2704 (2023). 37235647 10.1126/sciadv.add2704 PMC10219586

[51] Chandra, A. & Skinner, J. *Geography and Racial Health Disparities* (National Bureau of Economic Research, 2003); 10.3386/w9513

[52] Suriyakumar, V. M., Ghassemi, M. & Ustun, B. When personalization harms: reconsidering the use of group attributes in prediction. In *Proc. 40th International Conference on Machine Learning* 33209–33228 (PMLR, 2023); https://proceedings.mlr.press/v202/suriyakumar23a.html

[53] Manski, C. F., Mullahy, J. & Venkataramani, A. S. Using measures of race to make clinical predictions: decision making, patient health, and fairness. *Proc. Natl Acad. Sci. USA* **120**, e2303370120 (2023). 37607231 10.1073/pnas.2303370120 PMC10469015

[54] Gulrajani, I. & Lopez-Paz, D. In search of lost domain generalization. In *Proc. of the International Conference on Learning Representations* (ICLR, 2021); https://openreview.net/pdf?id=lQdXeXDoWtI

[55] Zhang, H. et al. An empirical framework for domain generalization in clinical settings. In *Proc. of the Conference on Health, Inference, and Learning* 279–290 (Association for Computing Machinery, 2021); 10.1145/3450439.3451878

[56] Branchaud-Charron, F., Atighehchian, P., Rodríguez, P., Abuhamad, G. & Lacoste, A. Can active learning preemptively mitigate fairness issues? Preprint at https://arxiv.org/abs/2104.06879 (2021).

[57] Artificial intelligence and machine learning (AI/ML)-enabled medical devices. *US Food & Drug Administration* https://www.fda.gov/medical-devices/software-medical-device-samd/artificial-intelligence-and-machine-learning-aiml-enabled-medical-devices (2022).

[58] Koh, P. W. et al. WILDS: a benchmark of in-the-wild distribution shifts. In *Proc. 38th**International Conference on Machine Learning* 5637–5664 (PMLR, 2021); https://cs.stanford.edu/people/jure/pubs/wilds-icml21.pdf

[59] Yang, Y., Zhang, H., Katabi, D. & Ghassemi, M. Change is hard: a closer look at subpopulation shift. In *Proc. 40th International Conference on Machine Learning* 39584–39622 (Association for Computing Machinery, 2023); https://proceedings.mlr.press/v202/yang23s/yang23s.pdf

[60] Mitchell, M. et al. Model cards for model reporting. In *Proc. of the Conference on Fairness, Accountability, and Transparency* 220–229 (Association for Computing Machinery, 2019); 10.1145/3287560.3287596

[61] Joint statement on enforcement efforts against discrimination and bias in automated systems. *Federal Trade Commission* https://www.ftc.gov/legal-library/browse/cases-proceedings/public-statements/joint-statement-enforcement-efforts-against-discrimination-bias-automated-systems (2023).

[62] Gallifant, J. et al. Disparity dashboards: an evaluation of the literature and framework for health equity improvement. *Lancet Digit. Health* **5**, e831–e839 (2023). 37890905 10.1016/S2589-7500(23)00150-4 PMC10639125

[63] Zhang, H. et al. Improving the fairness of chest x-ray classifiers. In *Proc. of the**Conference on Health, Inference, and Learning* Vo. 174, 204–233 (PMLR, 2022); https://proceedings.mlr.press/v174/zhang22a/zhang22a.pdf

[64] Zietlow, D. et al. Leveling down in computer vision: Pareto inefficiencies in fair deep classifiers. In *Proc. of the IEEE/CVF Conference on Computer Vision and Pattern Recognition* 10410–10421 (IEEE, 2022); https://openaccess.thecvf.com/content/CVPR2022/papers/Zietlow_Leveling_Down_in_Computer_Vision_Pareto_Inefficiencies_in_Fair_Deep_CVPR_2022_paper.pdf

[65] Petersen, E., Holm, S., Ganz, M. & Feragen, A. The path toward equal performance in medical machine learning. *Patterns* **4**, 100790 (2023). 37521051 10.1016/j.patter.2023.100790 PMC10382979

[66] Petersen, E., Ferrante, E., Ganz, M. & Feragen, A. Are demographically invariant models and representations in medical imaging fair? Preprint at https://arxiv.org/abs/2305.01397 (2023).

[67] Martinez, N., Bertran, M. & Sapiro G. Minimax Pareto fairness: a multi objective perspective. In *Proc. of the International Conference on Machine Learning* Vol. 119, 6755–6764 (PMLR, 2020); https://proceedings.mlr.press/v119/martinez20a.html PMC7912461 33644764

[68] Paulus, J. K. & Kent, D. M. Predictably unequal: understanding and addressing concerns that algorithmic clinical prediction may increase health disparities. *npj Digit. Med.* **3**, 99 (2020). 10.1038/s41746-020-0304-9 PMC7393367 32821854

[69] Movva, R. et al. Coarse race data conceals disparities in clinical risk score performance. *In Proc. of the 8th Machine Learning for Healthcare Conference*. https://proceedings.mlr.press/v219/movva23a.html 443-472 (PMLR, 2023).

[70] Johnson, A. E. W. et al. MIMIC-IV, a freely accessible electronic health record dataset. *Sci. Data* **10**, 1 (2023). 36596836 10.1038/s41597-022-01899-x PMC9810617

[71] Fanni, S. C. et al. Artificial intelligence-based software with CE mark for chest X-ray interpretation: opportunities and challenges. *Diagnostics (Basel)* **13**, 2020 (2023). 37370915 10.3390/diagnostics13122020 PMC10297610

[72] Lipton, Z. C., Elkan, C. & Naryanaswamy, B. Optimal thresholding of classifiers to maximize F1 measure. *Mach. Learn. Knowl. Discov. Databases*10.1007/978-3-662-44851-9_15 (2014). 10.1007/978-3-662-44851-9_15 PMC4442797 26023687

[73] Guo, C., Pleiss, G., Sun, Y. & Weinberger, K. Q. On calibration of modern neural networks. In *Proc. of the 34th International Conference on Machine Learning* 1321–1330 (PMLR, 2017); https://proceedings.mlr.press/v70/guo17a/guo17a.pdf

[74] Kuppers, F., Kronenberger, J., Shantia, A. & Haselhoff A. Multivariate confidence calibration for object detection. In *Proc. of the 2020 IEEE/CVF Conference on Computer Vision and Pattern Recognition Workshops (CVPRW)* 1322–1330 (IEEE, 2020); 10.1109/CVPRW50498.2020.00171

[75] Barocas, S., Hardt, M. & Narayanan, A. *Fairness and Machine Learning: Limitations and Opportunities* (MIT Press, 2019).

[76] Pfohl, S. et al. Net benefit, calibration, threshold selection, and training objectives for algorithmic fairness in healthcare. In *Proc. of the 2022 ACM Conference on Fairness, Accountability, and Transparency* 1039–1052 (Association for Computing Machinery, 2022); 10.1145/3531146.3533166

[77] Bernhardt, M., Jones, C. & Glocker, B. Potential sources of dataset bias complicate investigation of underdiagnosis by machine learning algorithms. *Nat. Med.* **28**, 1157–1158 (2022). 35710993 10.1038/s41591-022-01846-8

[78] Aickin, M. & Gensler, H. Adjusting for multiple testing when reporting research results: the Bonferroni vs Holm methods. *Am. J. Public Health* **5**, 726–728 (1996). 10.2105/ajph.86.5.726 PMC1380484 8629727

[79] Wang, Z., Wu, Z., Agarwal, D. & Sun, J. MedCLIP: contrastive learning from unpaired medical images and text. In *Proc. of the 2022 Conference on Empirical Methods in Natural Language Processing* (eds Goldberg, Y. et al.) 3876–3887 (Association for Computational Linguistics, 2022); 10.18653/v1/2022.emnlp-main.256 10.18653/v1/2022.emnlp-main.256 PMC11323634 39144675

[80] Tiu, E. et al. Expert-level detection of pathologies from unannotated chest X-ray images via self-supervised learning. *Nat. Biomed. Eng.* **6**, 1399–1406 (2022). 36109605 10.1038/s41551-022-00936-9 PMC9792370

[81] Rabanser, S., Günnemann, S. & Lipton, Z. Failing loudly: an empirical study of methods for detecting dataset shift. In *Proc. 33rd Conference on Neural Information Processing Systems* (NeurIPS, 2019); https://proceedings.neurips.cc/paper_files/paper/2019/file/846c260d715e5b854ffad5f70a516c88-Paper.pdf

[82] Deng, J. et al. Imagenet: a large-scale hierarchical image database. In *Proc. of the 2009 IEEE Conference on Computer Vision and Pattern Recognition* 248–255 (IEEE, 2009); 10.1109/CVPR.2009.5206848

[83] Bergstra, J. & Bengio, Y. Random search for hyper-parameter optimization. *J. Mach. Learn. Res.* **13**, 281–305 (2012).

[84] Pedregosa, F. et al. Scikit-learn: machine learning in Python. *J. Mach. Learn. Res.* **12**, 2825–2830 (2011).

