# Supplementary material for: The limits of fair medical imaging AI in real-world generalization
Source: Nat Med. 2024 Jun 28;30(10):2838–48. doi: 10.1038/s41591-024-03113-4 (PMC11485237; doi:10.1038/s41591-024-03113-4)
Supplement: Supplementary file 1 — Supplementary Tables 1–4, Figs. 1–4 and Note 1. [file 41591_2024_3113_MOESM1_ESM.pdf]

nature medicine

Supplementary information https://doi.org/10.1038/s41591-024-03113-4

The limits of fair medical imaging AI in real-world generalization

In the format provided by the authors and unedited

Supplementary Information

Supplementary Table 1. Prevalence shifts quantification in the ID settings across different tasks on MIMIC-CXR. We extend the quantification of prevalence shifts P(Y|A) in Extended Data Table 2 to different tasks, including (**a**) No Finding, (**b**) Cardiomegaly, (**c**) Effusion, and (**d**) Pneumothorax. Prevalence shift P(Y|A) is derived using the total variational distance between the probability distributions of Y conditioned on different groups. p-values were computed using a two-sided proportion z-test. All p-values were adjusted for multiple testing using Bonferroni correction[78].

a No Finding

Attribute	Group 1	Group 2	Distance	p value†
Sex	Female	Male	0.054	***
Age	40-60	18-40	0.187	***
	80-100	40-60	0.221	***
	80-100	60-80	0.083	***
	80-100	18-40	0.408	***
	60-80	40-60	0.138	***
	60-80	18-40	0.325	***
Race	White	Black	0.097	***
	White	Other	0.176	***
	White	Asian	0.005	not significant
	Black	Other	0.079	***
	Black	Asian	0.092	***
	Asian	Other	0.171	***

† Bonferroni correction for multiple testing. *** indicates $p < 0.001$.

b Cardiomegaly

Attribute	Group 1	Group 2	Distance	p value†
Sex	Female	Male	0.005	not significant
Age	40-60	18-40	0.048	***
	80-100	40-60	0.119	***
	80-100	60-80	0.055	***
	80-100	18-40	0.167	***
	60-80	40-60	0.063	***
	60-80	18-40	0.112	***
Race	White	Black	0.026	***
	White	Other	0.041	***
	White	Asian	0.017	not significant
	Black	Other	0.067	***
	Black	Asian	0.009	not significant
	Asian	Other	0.057	***

† Bonferroni correction for multiple testing. *** indicates $p < 0.001$.

c Effusion

Attribute	Group 1	Group 2	Distance	p value†
Sex	Female	Male	0.023	***
Age	40-60	18-40	0.069	***
	80-100	40-60	0.156	***
	80-100	60-80	0.066	***
	80-100	18-40	0.225	***
	60-80	40-60	0.090	***
	60-80	18-40	0.159	***
Race	White	Black	0.108	***
	White	Other	0.116	***
	White	Asian	0.012	not significant
	Black	Other	0.008	not significant
	Black	Asian	0.120	***
	Asian	Other	0.128	***

† Bonferroni correction for multiple testing. *** indicates $p < 0.001$.

d Pneumothorax

Attribute	Group 1	Group 2	Distance	p value†
Sex	Female	Male	0.012	***
Age	40-60	18-40	0.008	not significant
	80-100	40-60	0.000	not significant
	80-100	60-80	0.009	*
	80-100	18-40	0.009	not significant
	60-80	40-60	0.009	***
	60-80	18-40	0.000	not significant
Race	White	Black	0.021	***
	White	Other	0.015	***
	White	Asian	0.009	not significant
	Black	Other	0.006	not significant
	Black	Asian	0.030	***
	Asian	Other	0.024	***

† Bonferroni correction for multiple testing. *** indicates $p < 0.001$.

Supplementary Table 2. Distribution shifts quantification in the ID settings using a different foundation model as encoder on MIMIC-CXR. We extend the quantification of prevalence and representation shifts in Extended Data Table 2 using a different foundation model (i.e., CheXzero[80]). Prevalence shift P(Y|A) is derived using the total variational distance between the probability distributions of Y conditioned on different groups. P-values were computed using a two-sided proportion z-test. Representation shift P(X|A) is derived using the MMD distance on specific subgroup between ID and OOD datasets[81]. p-values were computed using a two-sided permutation test using this distance as the test statistic[81]. All p-values were adjusted for multiple testing using Bonferroni correction[78].

a Shifts in Y: $P(Y|A=a_1)$ vs. $P(Y|A=a_2)$

Attribute	Group 1	Group 2	Distance	p value†
Sex	Female	Male	0.054	***
Age	40-60	18-40	0.187	***
	80-100	40-60	0.221	***
	80-100	60-80	0.083	***
	80-100	18-40	0.408	***
	60-80	40-60	0.138	***
	60-80	18-40	0.325	***
Race	White	Black	0.097	***
	White	Other	0.176	***
	White	Asian	0.005	not significant
	Black	Other	0.079	***
	Black	Asian	0.092	***
	Asian	Other	0.171	***

† Bonferroni correction for multiple testing. *** indicates $p < 0.001$.

b Shifts in X: $P(X|A=a_1)$ vs. $P(X|A=a_2)$

Attribute	Group 1	Group 2	Distance	p value†
Sex	Female	Male	0.003	***
Age	40-60	18-40	0.016	***
	80-100	40-60	0.020	***
	80-100	60-80	0.004	***
	80-100	18-40	0.063	***
	60-80	40-60	0.007	***
	60-80	18-40	0.040	***
Race	White	Black	0.004	***
	White	Other	0.008	***
	White	Asian	0.002	***
	Black	Other	0.004	***
	Black	Asian	0.004	***
	Asian	Other	0.006	***

† Bonferroni correction for multiple testing. *** indicates $p < 0.001$.

Supplementary Table 3. Distribution shifts quantification in the OOD settings using a different foundation model as encoder. We extend the quantification of different shifts in Extended Data Table 3 using a different foundation model (i.e., CheXzero[80]). **a**, Label shift P(Y) is derived using the total variational distance between the probability distributions of Y between ID and OOD datasets. p-values were computed using a two-sided proportion z-test. **b**, Covariate shift P(X) is derived by first encoding input into representations from frozen CheXzero[80] then computing the Mean Maximum Discrepancy (MMD) distance with a Gaussian kernel[81] between ID and OOD datasets. p-values were computed using a two-sided permutation test using this distance as the test statistic[81]. **c**, Prevalence shift P(Y|A=a) is derived using the total variational distance conditioned on specific subgroup between ID and OOD datasets. p-values were computed using a two-sided proportion z-test. **d**, Representation shift P(X|A=a) is derived using the MMD distance on specific subgroup between ID and OOD datasets[81]. p-values were computed using a two-sided permutation test using this distance as the test statistic[81]. All p-values were adjusted for multiple testing using Bonferroni correction[78].

a Shifts in Y: $P_{\text{ID}}(Y)$ vs. $P_{\text{OOD}}(Y)$

ID dataset	OOD dataset	Distance	p value†
MIMIC	CheXpert	0.299	***
	NIH	0.142	***
	PadChest	0.045	***
	VinDr	0.014	not significant
	Combined	0.109	***
CheXpert	MIMIC	0.299	***
	NIH	0.442	***
	PadChest	0.254	***
	VinDr	0.313	***
	Combined	0.191	***

† Bonferroni correction for multiple testing. *** indicates $p < 0.001$.

b Shifts in X: $P_{\text{ID}}(X)$ vs. $P_{\text{OOD}}(X)$

ID dataset	OOD dataset	Distance	p value†
MIMIC	CheXpert	0.017	***
	NIH	0.014	***
	PadChest	0.302	***
	VinDr	0.030	***
	SIIM	0.011	***
CheXpert	MIMIC	0.017	***
	NIH	0.036	***
	PadChest	0.306	***
	VinDr	0.059	***
	SIIM	0.023	***

† Bonferroni correction for multiple testing. *** indicates $p < 0.001$.

c Shifts in $Y|A$: $P_{\text{ID}}(Y|A=a)$ vs. $P_{\text{OOD}}(Y|A=a)$‡

Attribute	Group	Distance	p value
Sex	Female	0.326	***
	Male	0.274	***
Age	80-100	0.201	***
	60-80	0.255	***
	40-60	0.338	***
	18-40	0.443	***
Race	White	0.256	***
	Black	0.324	***
	Asian	0.252	***
	Other	0.414	***

‡ ID and OOD datasets here are MIMIC and CheXpert, respectively.

d Shifts in $X|A$: $P_{\text{ID}}(X|A=a)$ vs. $P_{\text{OOD}}(X|A=a)$‡

Attribute	Group	Distance	p value
Sex	Female	0.326	***
	Male	0.274	***
Age	80-100	0.201	***
	60-80	0.255	***
	40-60	0.338	***
	18-40	0.443	***
Race	White	0.256	***
	Black	0.324	***
	Asian	0.252	***
	Other	0.414	***

‡ ID and OOD datasets here are MIMIC and CheXpert, respectively.

Supplementary Table 4. Direct demographic attribute prediction using an ERM model for the ISIC dataset. The AUROC values are averaged across 3 independent runs.

	**Sex**			**Age**			
	**Overall**	Female	Male	**Overall**	18-40	40-60	60-80
AUROC	**0.857** ±0.007	0.857 ±0.007	0.857 ±0.007	**0.750** ±0.005	0.806 ±0.008	0.657 ±0.006	0.787 ±0.003

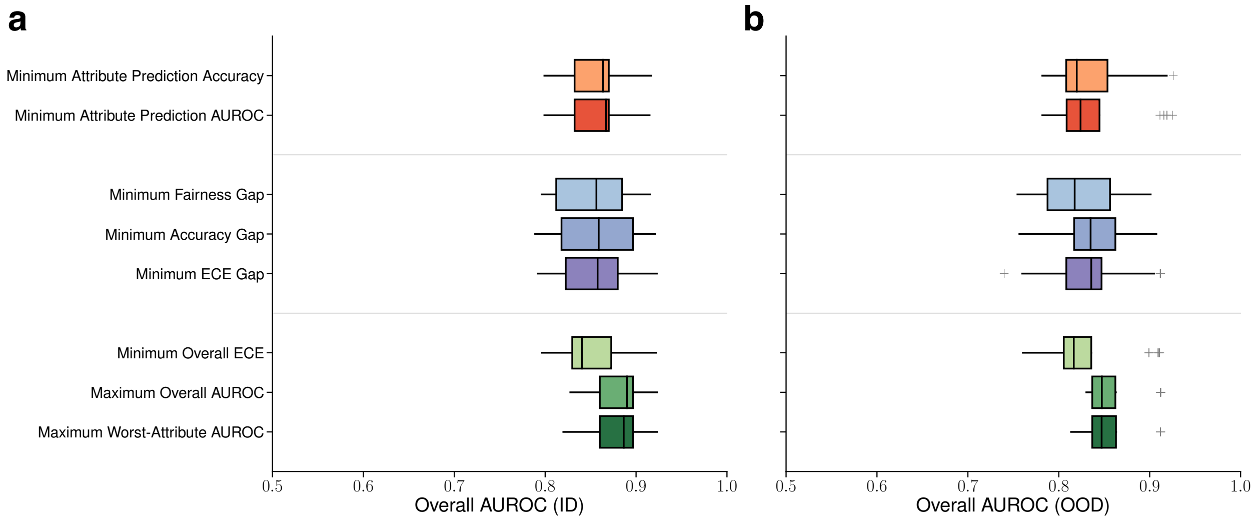

Supplementary Figure 1. OOD performance of models with different model selection criteria and for different algorithms. a, We vary the in-distribution model selection criteria, and compare the selected model against the oracle which chooses the model that is most fair OOD. We plot the overall AUROC for the ID test set, averaged across 42 combinations of OOD dataset, task, and attribute. We use non-parametric bootstrap sampling (n=1,000) to define the bootstrap distribution for the metric. **b,** We use the same criteria, and plot the overall AUROC for the OOD test set. On each box, the central line indicates the median, and the bottom and top edges of the box indicate the 25th and 75th percentiles, respectively. The whiskers extend to 1.5 times the interquartile range. Points beyond the whiskers are plotted individually using the + symbol.

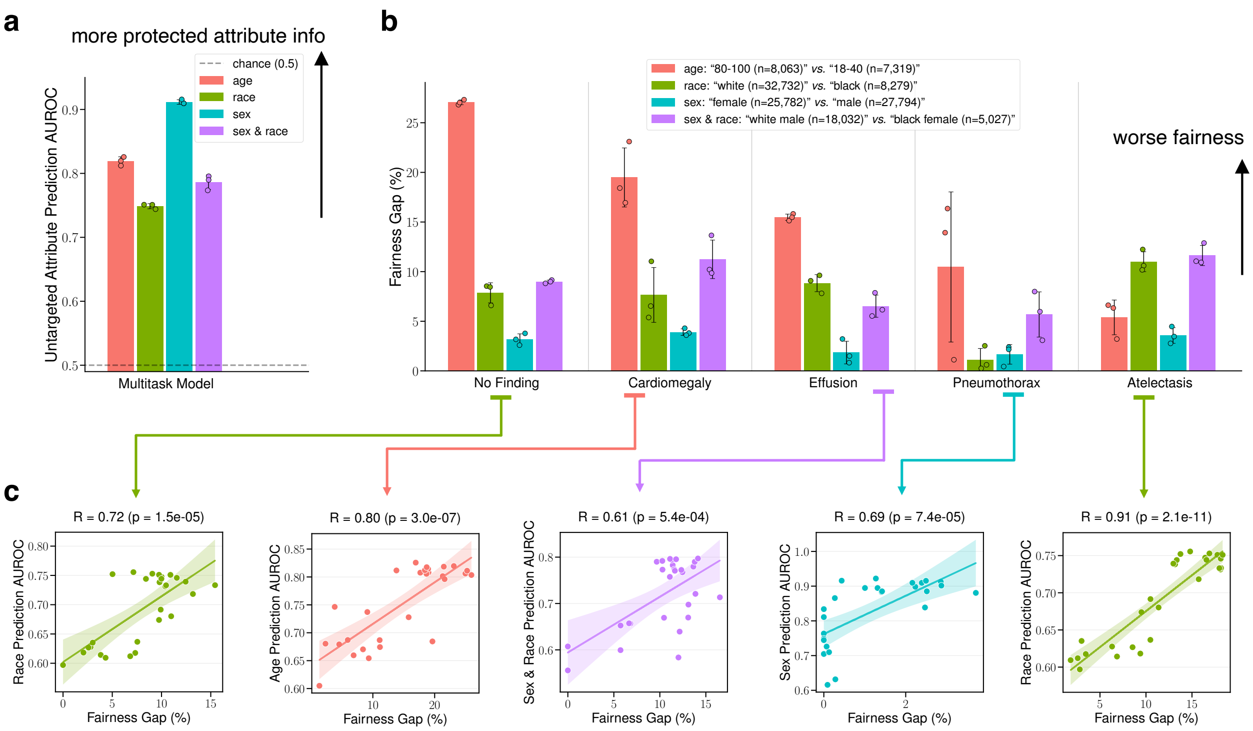

Supplementary Figure 2. Medical imaging models trained in a multilabel setup encode sensitive attributes and are unfair across subgroups. a, The area under the ROC curve (AUROC) of demographic attribute prediction from frozen representations for the best multilabel ERM model. We train multilabel ERM models on MIMIC-CXR to predict fourteen different tasks. The representations encode demographic attributes to a high degree. Each bar and its error bar indicate the mean and standard deviation across 3 independent runs. **b,** The fairness gap, as defined by the FPR gap for No Finding, and the FNR gap for all other tasks. The multilabel model exhibits high fairness gaps, especially between age groups. Each bar and its error bar indicate the mean and standard deviation across 3 independent runs. **c,** The correlation between attribute prediction performance and fairness for the multilabel model. We exclude models with suboptimal performance, i.e., with an overall validation AUROC below 0.7. The attribute prediction AUROC shows a high correlation with the fairness gap (No Finding, race: R=0.72, p=1.5e-05; Cardiomegaly, age: R=0.80, p=3.0e-07; Effusion, sex & race: R=0.61, p=5.4e-04; Pneumothorax, sex: R=0.69, p=7.4e-05; Atelectasis, race: R=0.91, p=2.1e-11; all using two-sided t-test). The center line and the shadow denote the mean and 95% CI, respectively.

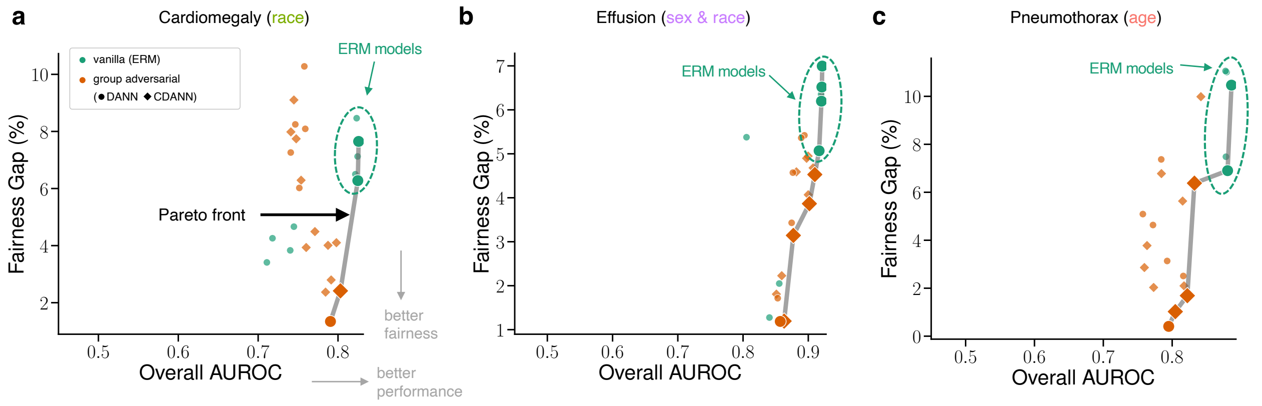

Supplementary Figure 3. Algorithms for removing demographic shortcuts mitigate in-distribution fairness gaps and maintain performance, using multilabel model training. a, b, c, Trade-off between the fairness gap and overall AUROC for all trained multilabel models. Each plot represents a specific disease prediction task (e.g., Cardiomegaly) with a specific attribute (e.g., race). In each case, we plot the Pareto front, the best achievable fairness gap with a minimum constraint on the performance.

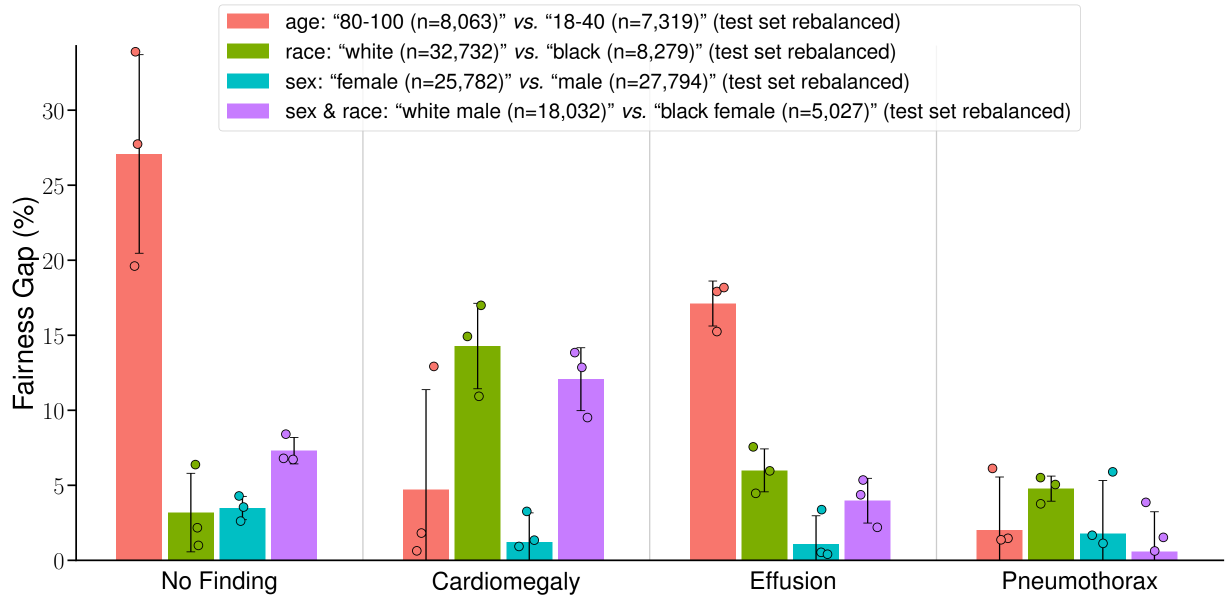

Supplementary Figure 4. The fairness gap for ERM models on the MIMIC dataset with test set rebalancing. We balance the test set across different attributes and the disease of interest, such that it eliminates prevalence shift in the test set. Compared to Fig. 2b, fairness gaps reduce across settings, yet still exist after test set rebalancing. Each bar and its error bar indicate the mean and standard deviation across 3 independent runs.

Supplementary Note 1. Checklist for supervised clinical ML study

**Before paper submission**			
**Study design (Part 1)**	**Completed: page number**		**Notes if not completed**
The clinical problem in which the model will be employed is clearly detailed in the paper.	☑	2, 3	
The research question is clearly stated.	☑	3	
The characteristics of the cohorts (training and test sets) are detailed in the text.	☑	4, 22, 32, 35, 36	
The cohorts (training and test sets) are shown to be representative of real-world clinical settings.	☑	4, 22	Note: All of our datasets are real clinical images released publicly by hospitals and research institutions.
The state-of-the-art solution used as a baseline for comparison has been identified and detailed.	☑	6-7, 26-27	
**Data and optimization (Parts 2, 3)**	**Completed: page number**		**Notes if not completed**
The origin of the data is described and the original format is detailed in the paper.	☑	30-31	
Transformations of the data before it is applied to the proposed model are described.	☑	22-23	
The independence between training and test sets has been proven in the paper.	☑	22	
Details on the models that were evaluated and the code developed to select the best model are provided.	☑	26-27	
Is the input data type structured or unstructured?	☐ Structured		☑ Unstructured
**Model performance (Part 4)**	**Completed: page number**		**Notes if not completed**
The primary metric selected to evaluate algorithm performance (eg: AUC, F-score, etc) including the justification for selection, has been clearly stated.	☑	23-26	
The primary metric selected to evaluate the clinical utility of the model (eg PPV, NNT, etc) including the justification for selection, has been clearly stated.	☑	23-26	
The performance comparison between baseline and proposed model is presented with the appropriate statistical significance.	☑	18, 29	

**Model Examination (Parts 5)**	**Completed: page number**		**Notes if not completed**
Examination Technique 1[a]	☑	7-9	
Examination Technique 2[a]	☑	15-16	
A discussion of the relevance of the examination results with respect to model/algorithm performance is presented.	☑	9-12, 19-21	
A discussion of the feasibility and significance of model interpretability at the case level if examination methods are uninterpretable is presented.	☑	15-16, 41	
A discussion of the reliability and robustness of the model as the underlying data distribution shifts is included.	☑	13-18, 19-21, 40	
**Reproducibility (Part 6): choose appropriate tier of transparency**			**Notes**
Tier 1: complete sharing of the code		☑	Code that supports the findings of this study is publicly available with an open-source license at https://github.com/YyzHarry/shortcut-ood-fairness.
Tier 2: allow a third party to evaluate the code for accuracy/fairness; share the results of this evaluation		☐	
Tier 3: release of a virtual machine (binary) for running the code on new data without sharing its details		☐	
Tier 4: no sharing		☐	

[a] Common examination approaches based on study type: for studies involving exclusively structured data, coefficients and sensitivity analysis are often appropriate; for studies involving unstructured data in the domains of image analysis or natural language processing, saliency maps (or equivalents) and sensitivity analyses are often appropriate. Select 2 from this list or chose an appropriate technique, document each technique used on the appropriate line above.
