# Supplementary material for: The limits of fair medical imaging AI in real-world generalization
Source: Nat Med. 2024 Jun 28;30(10):2838–48. doi: 10.1038/s41591-024-03113-4 (PMC11485237; doi:10.1038/s41591-024-03113-4)
Supplement: Supplementary file 2 — Reporting Summary [file 41591_2024_3113_MOESM2_ESM.pdf]

nature portfolio

Corresponding author(s): Yuzhe Yang

Last updated by author(s): May 30, 2024

Reporting Summary

Nature Portfolio wishes to improve the reproducibility of the work that we publish. This form provides structure for consistency and transparency in reporting. For further information on Nature Portfolio policies, see our Editorial Policies and the Editorial Policy Checklist.

Statistics

For all statistical analyses, confirm that the following items are present in the figure legend, table legend, main text, or Methods section.

n/a	Confirmed	
☐	☒	The exact sample size (*n*) for each experimental group/condition, given as a discrete number and unit of measurement
☐	☒	A statement on whether measurements were taken from distinct samples or whether the same sample was measured repeatedly
☐	☒	The statistical test(s) used AND whether they are one- or two-sided<br>*Only common tests should be described solely by name; describe more complex techniques in the Methods section.*
☐	☒	A description of all covariates tested
☐	☒	A description of any assumptions or corrections, such as tests of normality and adjustment for multiple comparisons
☐	☒	A full description of the statistical parameters including central tendency (e.g. means) or other basic estimates (e.g. regression coefficient) AND variation (e.g. standard deviation) or associated estimates of uncertainty (e.g. confidence intervals)
☐	☒	For null hypothesis testing, the test statistic (e.g. *F*, *t*, *r*) with confidence intervals, effect sizes, degrees of freedom and *P* value noted<br>*Give P values as exact values whenever suitable.*
☒	☐	For Bayesian analysis, information on the choice of priors and Markov chain Monte Carlo settings
☒	☐	For hierarchical and complex designs, identification of the appropriate level for tests and full reporting of outcomes
☐	☒	Estimates of effect sizes (e.g. Cohen's *d*, Pearson's *r*), indicating how they were calculated

Our web collection on statistics for biologists contains articles on many of the points above.

Software and code

Policy information about availability of computer code

Data collection	No software was used for data collection.
Data analysis	Code was written using Python version 3.9 (Python Software Foundation), with pytorch v1.12.0 and scikit-learn v1.2.2. Code is available for non-commercial research purpose, and is publicly available with an open-source license at https://github.com/YyzHarry/shortcut-ood-fairness.

For manuscripts utilizing custom algorithms or software that are central to the research but not yet described in published literature, software must be made available to editors and reviewers. We strongly encourage code deposition in a community repository (e.g. GitHub). See the Nature Portfolio guidelines for submitting code & software for further information.

Data

Policy information about availability of data

All manuscripts must include a data availability statement. This statement should provide the following information, where applicable:

- Accession codes, unique identifiers, or web links for publicly available datasets
- A description of any restrictions on data availability
- For clinical datasets or third party data, please ensure that the statement adheres to our policy

All datasets used in this study are publicly available. The MIMIC-CXR and VinDr-CXR datasets are available from PhysioNet after the completion of a data use agreement and a credentialing procedure. The CheXpert dataset, along with associated race labels, is available from the Stanford AIMI website. The ChestX-ray14

(NIH) dataset is available to download from the National Institute of Health Clinical Center. The PadChest dataset can be downloaded from the Medical Imaging Databank of the Valencia Region. The SIIM-ACR Pneumothorax Segmentation dataset can be downloaded from its Kaggle contest page. The ISIC 2020 dataset can be downloaded from the SIIM-ISIC Melanoma Classification Challenge page. The ODIR dataset can be obtained from the ODIR 2019 challenge hosted by Grand Challenges.

Research involving human participants, their data, or biological material

Policy information about studies with human participants or human data. See also policy information about sex, gender (identity/presentation), and sexual orientation and race, ethnicity and racism.

Reporting on sex and gender

For all datasets, self-reported sex was used for fairness analyses. The datasets used in this study have sex distributions publicly reported (Table 1, Extended Data Figure la & 2a). We report the results stratified by sex to assess the model performance on different subgroups, and explore the ability of the trained models to predict sex from medical images in the datasets used.

- MIMIC-CXR: 170,698 images (female) and 186,469 images (male)
- CheXpert: 90,576 images (female) and 132,216 images (male)
- NIH: 48,780 images (female) and 63,340 images (male)
- SIIM: 5,161 images (female) and 6,421 images (male)
- PadChest: 72,858 images (female) and 71,620 images (male)
- VinDr: 2,741 images (female) and 3,613 images (male)
- ISIC: 15,978 images (female) and 17,080 images (male)
- ODIR: 3,196 images (female) and 3,766 images (male)

Reporting on race, ethnicity, or other socially relevant groupings

Self-reported race was used for fairness analyses in MIMIC-CXR and CheXpert. Race information is not available in NIH, SIIM, PadChest, VinDr, ISIC, and ODIR. We report the results stratified by race to assess the model performance on different subgroups, and explore the ability of the trained models to predict race from medical images in the datasets used.

- MIMIC-CXR: 11,121 images (Asian), 55,611 images (Black), 218,037 images (White), and 72,398 images (Others)
- CheXpert: 23,298 images (Asian), 11,970 images (Black), 125,624 images (White), and 61,900 images (Others)

Population characteristics

See above, as well as Table 1 in the paper.

Recruitment

No participants were recruited for this retrospective study.

Ethics oversight

The study used data collected retrospectively. Approval of a study protocol was not needed.

Note that full information on the approval of the study protocol must also be provided in the manuscript.

Field-specific reporting

Please select the one below that is the best fit for your research. If you are not sure, read the appropriate sections before making your selection.

☒ Life sciences ☐ Behavioural & social sciences ☐ Ecological, evolutionary & environmental sciences

For a reference copy of the document with all sections, see nature.com/documents/nr-reporting-summary-flat.pdf

Life sciences study design

All studies must disclose on these points even when the disclosure is negative.

Sample size

We utilized seven datasets from the radiology domain, which encompass the set of publicly available chest X-ray datasets that have been widely used in prior work. These datasets span three continents, and allow us to evaluate model performance and fairness under real-world distribution shifts. In addition, we utilized one dataset each from the ophthalmology and dermatology domains, to validate our in-distribution findings on other medical imaging modalities. Sample sizes are the following:

Radiology: In total, we used 854,493 chest X-ray images for evaluation, coming from 5 internationally-sourced datasets: MIMIC (357,167 images), CheXpert (222,792 images), NIH (112,120 images), SIIM (11,582 images), PadChest (144,478 images), and Vin Dr (6,354 images).
Ophthalmology: We used ophthalmology images from ODIR dataset (6,800 images).
Dermatology: We used dermatology images from ISIC dataset (32,259 images).

Data exclusions

For all datasets, we exclude samples where the corresponding patient has missing age or sex. For ODIR and ISIC, we drop samples from patients younger than 18 and older than 80 due to small sample sizes (i.e., smaller than 3% of the total dataset).

No other data were excluded.

Replication

The code and data used to evaluate the findings are publicly available (as detailed in the Data-availability and Code-availability statements), can be used to replicate the findings.

Randomization

Randomization was used to create the train, validation, and test sets for each of the datasets used in the study.

Blinding

As the study contained no human-subject evaluations or interventions, and only profiles computational models on a fixed retrospectively collected dataset, no blinding was necessary.

Reporting for specific materials, systems and methods

We require information from authors about some types of materials, experimental systems and methods used in many studies. Here, indicate whether each material, system or method listed is relevant to your study. If you are not sure if a list item applies to your research, read the appropriate section before selecting a response.

Materials & experimental systems

n/a	Involved in the study
☒	☐ Antibodies
☒	☐ Eukaryotic cell lines
☒	☐ Palaeontology and archaeology
☒	☐ Animals and other organisms
☒	☐ Clinical data
☒	☐ Dual use research of concern
☒	☐ Plants

Methods

n/a	Involved in the study
☒	☐ ChIP-seq
☒	☐ Flow cytometry
☒	☐ MRI-based neuroimaging

Plants

Seed stocks

Report on the source of all seed stocks or other plant material used. If applicable, state the seed stock centre and catalogue number. If plant specimens were collected from the field, describe the collection location, date and sampling procedures.

Novel plant genotypes

Describe the methods by which all novel plant genotypes were produced. This includes those generated by transgenic approaches, gene editing, chemical/radiation-based mutagenesis and hybridization. For transgenic lines, describe the transformation method, the number of independent lines analyzed and the generation upon which experiments were performed. For gene-edited lines, describe the editor used, the endogenous sequence targeted for editing, the targeting guide RNA sequence (if applicable) and how the editor was applied.

Authentication

Describe any authentication procedures for each seed stock used or novel genotype generated. Describe any experiments used to assess the effect of a mutation and, where applicable, how potential secondary effects (e.g. second site T-DNA insertions, mosiacism, off-target gene editing) were examined.
